# Development of machine learning models to prognosticate chronic shunt-dependent hydrocephalus after aneurysmal subarachnoid hemorrhage

**DOI:** 10.1007/s00701-020-04484-6

**Published:** 2020-07-08

**Authors:** Giovanni Muscas, Tommaso Matteuzzi, Eleonora Becattini, Simone Orlandini, Francesca Battista, Antonio Laiso, Sergio Nappini, Nicola Limbucci, Leonardo Renieri, Biagio R. Carangelo, Salvatore Mangiafico, Alessandro Della Puppa

**Affiliations:** 1 Neurosurgery Clinic, Department of Neuroscience, Psychology, Pharmacology and Child Health, Careggi University Hospital and University of Florence, Largo Piero Palagi 1, 50137 Florence, Italy; 2 Institute of Physics, Alma Mater Studiorum, University of Bologna, Bologna, Italy; 3 Interventional Neuroradiology Unit, Department of Neuroscience, Psychology, Pharmacology and Child Health, Careggi University Hospital and University of Florence, Florence, Italy; 4 Department of Neurosurgery, Le Scotte University Hospital, Siena, Italy

**Keywords:** Subarachnoid hemorrhage, Shunt-dependency, Hydrocephalus, Machine learning, Prognostic models

## Abstract

**Background:**

Shunt-dependent hydrocephalus significantly complicates subarachnoid hemorrhage (SAH), and reliable prognosis methods have been sought in recent years to reduce morbidity and costs associated with delayed treatment or neglected onset. Machine learning (ML) defines modern data analysis techniques allowing accurate subject-based risk stratifications. We aimed at developing and testing different ML models to predict shunt-dependent hydrocephalus after aneurysmal SAH.

**Methods:**

We consulted electronic records of patients with aneurysmal SAH treated at our institution between January 2013 and March 2019. We selected variables for the models according to the results of the previous works on this topic. We trained and tested four ML algorithms on three datasets: one containing binary variables, one considering variables associated with shunt-dependency after an explorative analysis, and one including all variables. For each model, we calculated AUROC, specificity, sensitivity, accuracy, PPV, and also, on the validation set, the NPV and the Matthews correlation coefficient (ϕ).

**Results:**

Three hundred eighty-six patients were included. Fifty patients (12.9%) developed shunt-dependency after a mean follow-up of 19.7 (± 12.6) months. Complete information was retrieved for 32 variables, used to train the models. The best models were selected based on the performances on the validation set and were achieved with a distributed random forest model considering 21 variables, with a ϕ = 0.59, AUC = 0.88; sensitivity and specificity of 0.73 (C.I.: 0.39–0.94) and 0.92 (C.I.: 0.84–0.97), respectively; PPV = 0.59 (0.38–0.77); and NPV = 0.96 (0.90–0.98). Accuracy was 0.90 (0.82–0.95).

**Conclusions:**

Machine learning prognostic models allow accurate predictions with a large number of variables and a more subject-oriented prognosis. We identified a single best distributed random forest model, with an excellent prognostic capacity (ϕ = 0.58), which could be especially helpful in identifying low-risk patients for shunt-dependency.

**Electronic supplementary material:**

The online version of this article (10.1007/s00701-020-04484-6) contains supplementary material, which is available to authorized users.

## Introduction

Shunt-dependent hydrocephalus is a significant complication of aneurysmal subarachnoid hemorrhage (SAH) affecting 11 to 39.6% of patients with aneurysmal SAH [1, 8, 13, 17, 27, 29, 35, 39, 49]. Several studies have tried to identify potential predictors of shunt-dependency to estimate prognosis, to offer the best treatment strategy by preventing complications associated with unnecessary treatment or delayed surgical intervention, and to reduce hospitalization and rehabilitation length and costs [1, 5, 8, 9, 11–13, 16, 17, 21–23, 25, 27, 29, 31, 33, 35, 36, 39, 43, 46–48, 50]. Among these variables, some have been proposed, like the patient’s age and gender [22, 23, 33, 35, 36, 47, 48], the neurological status at presentation (Hunt & Hess and WFNS scales) [12, 20–22, 25, 27, 47, 48], the amount of cisternal blood on the first CT scan (Fisher and BNI scales) [12, 16, 21–23, 25, 35, 47], the presence of acute hydrocephalus on the first CT scan and the need for external ventricular drain (EVD) [1, 12, 21–23, 25, 27, 33, 36, 43, 47, 48], the duration of EVD treatment [25, 48], aneurysms location and size [9, 22, 25, 33, 36, 47], the type of treatment for aneurysm exclusion (endovascular or surgical) [9, 25, 35, 36, 50], the onset and duration of posttreatment complications (i.e., fever and/or infections) [25, 27, 36, 47, 50], the duration of blood clearance detected on serial CT scans [29], and altered values of blood or cerebrospinal fluid (CSF) markers [27, 31, 46]. Some meta-analyses [11, 45, 47, 50] have synthesized this information, and scores for risk stratification have been proposed to be used in the clinical practice [5, 12, 13, 21, 27], whose validity, however, has not yet been confirmed on other cohorts.

Despite consistent associations between some variables and the development of shunt-dependency across studies, results differ on the role of other potentially important items, with the likely effect of scores over-emphasizing some variables while neglecting relevant ones. This could represent a limitation when risk scores built on these premises are used in clinical practice.

Modern standards of data analysis and prediction models rely on machine learning (ML), a branch of statistical analysis that is gaining more and more consideration in the medical field due to its excellent results and, more recently, also in neurosurgery [6, 10, 18, 34, 41, 42, 44]. ML consists of algorithm-based models with the ability to learn and perform tasks that are not explicitly programmed, to improve the performances with experience (i.e., when the model analyzes new data), and to work with a large amount of data and nonlinear associations, where classical statistical methods can show some limitations [6, 18, 38].

We aimed at testing the ability of machine learning models to predict the development of shunt-dependent hydrocephalus in aneurysmal SAH patients, intending to develop a prognostic model based on current data analysis standards, in order to reduce omission of potentially relevant variables and allow for better individual risk estimation.

## Methods

### Data collection and variables selection

Electronic files and radiological data of patients undergoing surgical or endovascular treatment for aneurysmal SAH at our institution between January 2013 and March 2019 were retrospectively consulted to collect information on variables potentially related to shunt-dependency, according to the results of previous works on this topic [1, 5, 7–9, 11–13, 16, 17, 21–23, 25, 27, 29, 33, 36, 39, 43, 46–48, 50]. Also, we expanded this information by including quantitative information on the neurological and general clinical status of patients at the time of the acute event (SAH), such as the Karnofsky performance status (KPS), the ASA physical status classification system, the modified rankin scale (mRS), and the National Institute of Health Stroke Scale (NIHSS). The variables considered are summarized in Table 1.

**Table 1:** Variables retrieved

Type	Variable
Patient-related	Age, gender, ASA, Karnofsky
Disease-related	Hunt-Hess, WFNS, GCS, NIHSS, supplementary motor NIHSS, mRS, clinical vasospasm, posttreatment fever, timing of fever onset and fever duration, meningitis, other infections, aneurysm location and max. diameter, multiple aneurysms, vasospasm
Radiological	Fisher, BNI, ICH or IVH, SAH and IVH sum score, BI, acute hydrocephalus on presentation, rebleeding
Treatment-related	Aneurysm treatment (endovascular or surgical), treatment timing, treatment complication, EVD insertion, and duration of EVD treatment

WFNS, World Federation of Neurosurgical Societies; GCS, Glasgow coma score; NIHSS, National Institute of Health Stroke Scale; mRS, modified rankin scale; ASA, American Society of Anesthesiologists; BNI, Barrow Neurological Institute; ICH, intracerebral hemorrhage; IVH, intraventricular hemorrhage; SAH, subarachnoid hemorrhage; BI, bicaudate index; EVD, external ventricular drain

Patients were subdivided according to their GCS at presentation into three groups to train the models: 12–15, 8–11, and < 8. We subdivided treatment timing into very early (< 6 h), early (6–12 h), late (12–24 h), and delayed (> 24 h). Patients were further dichotomized according to the duration of EVD permanence and fever in ≤ 5 days and > 5 days. Fever onset was dichotomized in early and delayed onset (cutoff: 7 days after treatment). The bicaudate index was measured at the first CT after symptoms onset, immediately after treatment and at 14 days (or at the last CT performed within 14 days from the acute event). Additionally, information on follow-up duration and the onset of shunt-dependency were retrieved.

### Treatment protocol

Patients with suspected aneurysmal SAH after skull CT and CT-angiography (CTA) referred to our institution were managed by a multidisciplinary team composed of neurosurgeons, endovascular neuroradiologists, and anesthesiologists who chose the most appropriate treatment on a case-by-case basis, taking into account the patient’s clinical status, age, and comorbidities, as well as the entity of SAH and the aneurysm location and morphology. In cases of imaging of insufficient quality of the CTA or unsatisfactory depiction, a digital subtraction angiography (DSA) of the intracranial vessels was performed.

After aneurysm occlusion (either endovascular or surgical), patients were transferred to the intensive care unit (ICU), the intermediate care (IMC) unit, or the neurosurgical ward according to the patients’ preoperative clinical status, age, comorbidities, entity of the subarachnoid hemorrhage, and performed treatment after the operating physician and the anesthesiologist had reached interdisciplinary consensus. In each case, patients underwent neurological and clinical monitoring for a minimum of 14 days after treatment, as well as routine transcranial Doppler studies for early detection of vasospasm. CT of the skull was performed immediately after treatment, whenever neurological deterioration occurred (pre- and postoperative), before sedation weaning (for intubated patients), before EVD removal, and before discharge.

### EVD insertion, weaning, and indications for permanent shunts

Indication for EVD was posed in patients with an acute neurological deterioration associated with radiological findings of acute hydrocephalus. In patients who were referred intubated to our institution, EVD was placed when the immediate pre- or postoperative scan showed acute hydrocephalus. In both cases, the reservoir was placed at such a height to drain 10 ml of CSF/h. Once the clinical and neurological status was stable, weaning began by increasing by 2 cm of H_2_O every 24–48 h until the absence of CSF drainage. Then, the drain was kept closed for 24–48 h, and if neurological status remained stable, a CT scan was performed. If no ventricle dilatation was documented, the EVD was removed. If neurological deterioration occurred during EVD weaning, a CT scan was performed, and in case of evident or suspected ventricle dilatation, the reservoir was open again to drain 10 ml of CSF/h. A new attempt of weaning was made following the same protocol, and if neurological deterioration with ventricle dilatation occurred a second time, the patient was deemed shunt-dependent.

Patients with poor clinical conditions or low GCS, in which recognizing neurological deterioration would have been more challenging during EVD weaning, were treated following the same protocol, and a CT scan was performed prior to each change of the reservoir height.

After discharge, if clinical conditions remained stable, patients underwent a clinical and radiological follow-up with CTA at 3 months, MR-angiography at 6 months, and DSA at 12 months. Other investigations were performed when deemed necessary, in case of suspected incomplete exclusion of the aneurysm or when neurological changes occurred. If ventricle enlargement was detected in association with neurological deterioration during follow-up, a permanent ventricular shunt was indicated.

### Statistical analysis, preprocessing, creation, and testing of models

Continuous variables are reported as mean with standard deviation, and categorical variables are expressed as percentages. Statistical analysis, data preprocessing, and graphics creation were performed with SPSS Statistics© 23 (IBM Corp. Armonk, NY, USA) and MATLAB R2020 (MathWorks Inc., Natick, MA, USA; https://www.mathworks. com). A Wilk-Shapiro test was used to assess normal distribution. We first conducted an exploratory statistical analysis, and a comparison of variables between shunt-dependent and non-shunt-dependent patients was performed with a *t* test for unpaired data for continuous variables and a *χ*^2^ test for categorical variables. A Bonferroni correction was used for multiple comparisons.

Before training machine learning models, missing variables for > 40% of patients were removed to avoid the significant influence of the imputation, as well as those patients with missing information on eight or more variables. Missing data were imputed with K-nearest neighbor imputation. Patients deceased before assessment of shunt-dependency were excluded, like those survived but missed on follow-up lacking information on the development of shunt-dependency.

ML models were trained using the open-source platform H_2_O (https://www.h2o.ai, Mountain View, CA, USA), which provides a package of scripts for ML algorithms whose parameter can be customized ad hoc. We used the web interface (H_2_O Flow) provided by the site running in Java™ (https://www.java.com, Oracle Corporation, Redwood, CA, USA). For our purposes, we tested four of the most frequently employed algorithms for supervised learning without knowing previously which one would be the most precise for our purposes: generalized linear modeling (GL), distributed random forest (DRF), gradient boosting machine (GBM), and deep learning (DL). The clean dataset was randomly split into training (75% of the patients) and validation set (25%). A 6-fold cross-validation was performed on the training set, before evaluating prediction performances on the validation set. Cross-validation is a resampling technique to obtain a more accurate and less biased estimate of how the model will score on previously unseen data. It consists of creating k samples (in our case, k = 6) of equal size from the training dataset, of which one is used as a validation set and the remaining as a training set. This process is repeated k times, using each of the subsamples once as a validation sample, and the results of all iterations are summarized by metrics mean and standard deviation. In our case, the area under the receiver operating characteristic curve (AUROC or AUC), accuracy, sensitivity, specificity, the positive predictive value (PPV), and the Matthews correlation coefficient were calculated. The Matthews correlation coefficient, or ϕ, is a measure of the quality of a binary classification used in machine learning, with scores ranging between + 1 identifying a perfect prediction and − 1 indicating total disagreement. A score equal to 0 means the model makes no better prediction than a random guess [4].

For each model, the algorithm parameters were customized and fine-tuned to obtain the optimized Matthews correlation coefficient. Also, the binarization threshold was chosen to maximize ϕ. Algorithms training was performed using logloss (logarithmic loss metric) as the stopping parameter: once the algorithm parameters are set by the operator, this procedure iterates the development of models of increasing complexity until the performance of the model decreases. The logloss evaluates how close the predicted values are to the actual ones. Values can be greater than or equal to 0, with 0 meaning that the model correctly predicts an event. For each model variable, importances were calculated, and recursive feature selection was performed by removing variables with lower coefficients stepwise until reaching optimal scores. Performances on the validation sets were synthesized in confusion matrices, and sensitivity, specificity, PPV and negative predictive value (NPV), and accuracy with 95% confidence intervals were calculated, along with the AUC and ϕ.

On both sets, calibration metrics were calculated as the Hosmer-Lemeshow goodness of fit test and as slope and intercept of the calibration curve.

## Results

During the considered period, 479 patients underwent treatment of aneurysmal SAH at our institution (mean age: 59 ± 13 years, 320 females [66.8%], mean follow-up: 19.7 ± 12.6 months). Variables retrieved were available in the following proportions: GCS at admission in 393 patients (82% of cases), Fisher score in 377 (78.5%), Hunt-Hess in 395 (82.3%), BNI in 152 (31.6%), WFNS in 392 (81.6%), ICH in 385 (80.2%), IVH in 388 (80.8%), treatment timing in 376 (78.3%), SAH sum score in 150 (31.2%), IVH sum score in 357 (74.4%), BI preoperative in 141 (29.3%), postoperative in 358 (74.6%), at 14 days in 170 (35.4%), at the last CT scan in 249 (51.9%), mRS on admission in 397 (82.7%), ASA class in 390 (81.2%), KPS score in 392 (81.6%), NIHSS score in 392 (81.6%), presence/absence of acute hydrocephalus in 330 (68.7%), need for EVD placement in 392 (81.6%), EVD duration—if EVD present—in 150 (31.2%), rebleeding in 393 (81.9%), aneurysm location in 400 (83.3%), multiple aneurysm in 389 (81%), aneurysm max. diameter in 370 (77.1%), treatment modality in 404 (84.2%), need for posttreatment ICU in 394 (82.1%), DCI in 388 (80.8%), treatment complication in 390 (81.2%), postoperative fever in 390 (81.2%), fever timing in 375 (78.1%), days with fever in 319 (66.5%), meningitis in 382 (79.6%) or other infections in 383 (79.8%), and development of shunt-dependency in 390 (81.2%).

After removing patients deceased before evaluation of the development of shunt-dependency (*n* = 29 [6%]), those with more than eight missing variables or missing information on the development of shunt-dependency (*n* = 64, 13.4%), and after eliminating all variables missing for more than 40% of patients, the clean dataset comprised 386 patients and 32 variables (Table 2).

**Table 2:** Variables used for the models creation

Variable; *n* = patients with available information (% of included patients)	Category	Total	Shunt –	Shunt +	*p* value
Sex; *n* = 386 (100%)	F	262 (67.9%)	222 (66.1%)	40 (80%)	0.05
	M	124 (32.1%)	114 (35.9%)	10 (20%)	
Age; *n* = 386 (100%)		58.9 (± 13.2)	58.5 (± 13.4)	61.6 (± 11)	0.1
GCS; *n* = 386 (100%)	12–15	294 (76.2%)	261 (77.7%)	41 (82%)	0.4
	8–11	31 (8%)	27 (8%)	4 (6%)	0.6
	< 8	61 (15.8%)	48 (14.3%)	6 (12%)	0.6
Fisher; *n* = 368 (95.3%)	0	1 (0.3%)	1 (0.3%)	0	0.7
	1	21 (5.7%)	20 (6.2%)	0	0.07
	2	52 (14.2%)	51 (16%)	1 (2.1%)	0.01
	3	108 (29.3%)	102 (32%)	6 (12.5%)	**0.005**
	4	186 (50.5%)	145 (45.4%)	41 (85.4%)	**< 0.0001**
Hunt-Hess; *n* = 386 (100%)	0	0	0	0	/
	1	128 (33.2%)	123 (36.6%)	5 (10%)	**< 0.0001**
	2	91 (23.6%)	82 (24.4%)	9 (18%)	0.5
	3	93 (24.1%)	69 (20.5%)	24 (48%)	**< 0.0001**
	4	28 (7.2%)	23 (6.9%)	5 (10%)	0.4
	5	46 (11.9%)	39 (11.6%)	7 (14%)	0.9
WFNS; *n* = 385 (99.7%)	1	201 (52.2%)	192 (57.3%)	9 (18%)	**<0.0001**
	2	59 (15.3%)	45 (13.4%)	14 (28%)	**0.008**
	3	17 (4.4%)	13 (3.9%)	4 (8%)	0.6
	4	57 (14.8%)	45 (13.4%)	12 (24%)	0.05
	5	51 (13.3%)	40 (12%)	11 (22%)	0.05
ICH; *n* = 378 (100%)	Yes	97 (25.7%)	76 (23.2%)	21 (42%)	0.1
	No	281 (74.3%)	252 (76.8%)	29 (58%)	
IVH; *n* = 382 (99%)	Yes	204 (53.4%)	162(48.6%)	42 (84%)	**< 0.0001**
	No	179 (46.6%)	171 (51.4%)	8 (16%)	
Treatment timing; *n* = 368 (95.3%)	<6 h	74 (20%)	62 (19.4%)	12 (24.5%)	0.5
	6–12 h	136 (37%)	114 (35.7%)	22 (44.9%)	0.3
	12–24 h	86 (23.4%)	75 (23.5%)	11 (22.4%)	0.7
	> 24 h	72 (19.6%)	68 (21.4%)	4 (8.2%)	0.1
IVH sum score; *n* = 349 (94.8%)		2.1 (± 2.9)	1.8 (± 2.7)	4.25 (± 3.3)	**< 0.0001**
Bicaudate index post-op; *n* = 354 (91.7%)		0.17 (± 0.06)	0.16 (± 0.06)	0.2 (± 0.05)	**0.0001**
mRS; *n* = 385 (99.7%)	0	80 (20.8%)	76 (22.7%)	4 (8%)	0.02
	1	31 (8.1%)	28 (8.6%)	3 (6%)	0.5
	2	87 (22.6%)	78 (23.3%)	9 (18%)	0.4
	3	51 (13.2%)	42 (12.5%)	9 (18%)	0.3
	4	61 (15.8%)	49 (14.6%)	12 (24%)	0.2
	5	75 (19.5%)	61 (18.3%)	13 (26%)	0.1
	6	0	0	0	/
ASA; *n* = 381 (98.7%)	1	128 (33.6%)	115 (34.6%)	13 (26.5%)	0.4
	2	128 (33.6%)	108 (32.5%)	20 (40.8%)	0.2
	3	79 (20.7%)	68 (20.5%)	11 (22.5%)	0.7
	4	10 (2.6%)	10 (3.1%)	0	0.2
	5	36 (9.5%)	31 (9.3%)	5 (10.2%)	0.5
KPS; *n* = 387 (100%)		64 (±29)	65 (±29)	53 (±27)	**0.004**
NIHSS; n = 387 (100%)		8 (±12)	7 (±12)	11 (±13)	0.03
Motor NIHSS; n = 387 (100%)		4 (±7)	4 (±7)	5 (±7)	0.2
Acute hydrocephalus; *n* = 325 (84.2%)	Yes	83 (25.5%)	59 (21%)	24 (54.3%)	**< 0.0001**
	No	242 (74.5%)	222 (81%)	20 (45.7%)	
EVD; n = 386 (100%)	Yes	115 (29.8%)	75 (22.3%)	40 (80%)	**< 0.0001**
	No	271 (70.2%)	261 (77.7%)	10 (20%)	
Days W. EVD; *n* = 386 (100%)	≤ 5 days	284 (73.6%)	271 (80.7%)	12 (24%)	**< 0.0001**
	> 5 days	102 (26.4%)	65 (19.3%)	38 (76%)	
Aneurysm location; *n* = 386 (100%)	AcoA	158 (40.9%)	134 (39.9%)	24 (48%)	0.05
	Carotid siphon	55 (14.2%)	48 (14.2%)	7 (14%)	0.8
	Pcom	32 (8.3%)	28 (8.4%)	4 (8%)	0.9
	MCA	78 (20.2%)	72 (21.4%)	6 (12%)	0.1
	ACA	12 (3.1%)	9 (2.7%)	3 (6%)	0.3
	PCA	1 (0.3%)	1 (0.3%)	0	0.5
	AICA	1 (0.3%)	1 (0.3%)	0	0.7
	PICA	10 (2.6%)	8 (2.3%)	2 (4%)	0.5
	Vertebral	7 (1.8%)	6 (1.8%)	1 (2%)	0.9
	Basilar	10 (2.6%)	9 (2.7%)	1 (2%)	0.9
	Pericallosal/callosomarginal	13 (3.5%)	12 (3.6%)	1 (2%)	0.6
	Ant. Choroidal	6 (1.5%)	5 (1.5%)	1 (2%)	0.3
	Ophthalmic	3 (0.7%)	3 (0.9%)	0	0.5
Rebleeding; *n* = 386 (100%)	Yes	38 (8.8%)	29 (8.7%)	9 (18%)	0.4
	No	348 (91.2%)	307 (91.3%)	41 (12%)	
Aneurysm max. diameter (mm); *n* = 386 (100%)		7.7 (± 5.9)	7.7 (± 6.1)	7.8 (± 4)	0.9
Treatment; *n* = 383 (99.2%)	Endovascular	320 (83.5%)	278 (83.5%)	42 (84%)	0.9
	Surgical	63 (16.5%)	55 (16.5%)	8 (16%)	
Posttreatment ICU; *n* = 386 (100%)	Yes	278 (72%)	229 (68.1%)	49 (98%)	**< 0.0001**
	No	108 (28%)	107 (31.9%)	1 (2%)	
DCI; *n* = 383 (99%)	Yes	130 (33.9%)	107 (32.1%)	23 (46%)	0.07
	No	253 (66.1%)	226 (67.9%)	27 (54%)	
Treatment complication; *n* = 382 (98.7%)	Yes	96 (25.1%)	81 (21.2%)	15 (30%)	0.5
	No	286 (74.9%)	249 (78.8%)	35 (70%)	
Multiple aneurysms; *n* = 378 (97.7%)	Yes	114 (30.6%)	94 (28.6%)	20 (40.8%)	0.2
	No	264 (69.4%)	235 (71.4%)	29 (59.2%)	
Fever; *n* = 375 (96.9%)	Yes	325 (86.6%)	279 (85.3%)	46 (95.8%)	0.6
	No	50 (13.4%)	48 (14.7%)	2 (4.2%)	
Fever onset; *n* = 310 (95.4%)*	< 7 days	294 (93.8%)	250 (89.6%)	44 (91.6%)	0.7
	> 7 days	16 (6.2%)	14 (10.4%)	2 (6.7%)	
Days w. fever; *n* = 386 (100%)	≤ 5 days	177 (45.8%)	168 (50%)	9 (18%)	**< 0.0001**
	> 5 days	209 (54.2%)	168 (50%)	41 (82%)	
Meningitis; *n* = 380 (98.4%)	Yes	18 (2.1%)	11 (3.3%)	7 (14.3%)	**0.001**
	No	362 (97.9%)	320 (96.7%)	42 (85.7%)	
Other infections; *n* = 381 (98.7%)	Yes	149 (39.1%)	114 (34.3%)	35 (71.4%)	**< 0.0001**
	No	232 (60.9%)	218 (65.7%)	14 (28.6%)	
**SDH;** ***n*** **= 386 (100%)**	**Yes**	**50 (12.9%)**			
	**No**	**336 (87.1%)**			

Significant association after Bonferroni correction are highlighted with bold digits. (*) Percentage of patients with fever

AcoA, anterior communicating artery; Pcom, posterior communicating artery; MCA, middle cerebral artery; ACA, anterior cerebral artery; PCA, posterior cerebral artery; AICA, anteroinferior cerebellar artery; PICA, posteroinferior cerebellar artery

The exploratory statistical analysis yielded significant association with the onset of shunt-dependency for the following variables: Fisher > 2 (*p* < 0.01), Hunt-Hess = 1 (*p* = 0.001, negative correlation) or 3 (*p* = 0.001, positive correlation), WFNS > 2 (*p* < 0.01), presence of IVH on admission (*p* < 0.001), higher preoperative IVH sum score (*p* = 0.001), higher postoperative BI (p = 0.001), lower KPS on admission (*p* = 0.004), acute hydrocephalus on admission (*p* < 0.001), need for EVD treatment perioperatively (*p* < 0.001), the permanence of EVD > 5 days (*p* < 0.001), the need for posttreatment ICU (*p* < 0.001), presence of postoperative fever lasting > 5 days (*p* < 0.001), and the postoperative development of meningitis (*p* = 0.001) or other infections (*p* < 0.001). See also Table 2 for details.

Performances after 6-fold cross-validation are summarized in Table 3. The highest accuracy and ϕ on the resampled training set was obtained with a DL algorithm with 31 variables (0.84 [± 0.07]) and 0.54 [± 0.1], respectively). However, the best performances on the validation set were reached with a DRF model including 21 variables (see Tables 4 and 5), with a correlation coefficient (ϕ) of 0.59 an AUC = 0.88, sensitivity and specificity of 0.73 (C.I.: 0.39–0.94) and 0.92 (C.I.: 0.84–0.97), respectively, PPV = 0.59 (0.38–0.77) and 0.96 (0.90–0.98). Accuracy was = 0.90 (0.82–0.95) (see also Table 4).

**Table 3:** Discrimination obtained after sixfold cross-validation on the training set (*n* = 296)

Algorithm	No. of variables included	AUC	Sensitivity	Specificity	PPV	Accuracy	ϕ
GL	12	0.81 (±0.09)	0.72 (±0.2)	0.82 (±0.1)	0.50 (±0.3)	0.82 (±0.1)	0.52 (±0.1)
DRF	21	0.85 (± 0.06)	0.78 (± 0.2)	0.84 (± 0.1)	0.50 (± 0.2)	0.84 (± 0.1)	0.53 (± 0.2)
GBM	28	0.74 (± 0.1)	0.68 (± 0.2)	0.86 (± 0.1)	0.58 (± 0.4)	0.83 (**±** 0.1)	0.51 (± 0.2)
DL	32	0.84 (± 0.07)	0.70 (± 0.2)	0.87 (± 0.1)	0.60 (± 0.3)	0.85 (± 0.1)	0.54 (± 0.1)

GL, generalized linear modeling; DRF, distributed random forest; GBM, gradient boosting machine; DL, deep learning; AUC, area under the receiver operating characteristic curve; PPV, positive predictive value

**Table 4:** Discrimination metrics on the validation set (*n* = 90). 95%-confidence intervals are reported in brackets

Algorithm	GL	DRF	GBM	DL
Binarization threshold	0.15	0.24	0.41	0.86
Sensitivity	0.73 (0.39–0.94)	0.73 (0.39–0.94)	1.0 (0.71–0.1)	0.45 (0.17–0.77)
Specificity	0.87 (0.78–0.94)	0.92 (0.84–0.97)	0.62 (0.50–0.73)	0.97 (0.1–1.0)
PPV	0.46 (0.30–0.63)	0.59 (0.38–0.77)	0.28 (0.23–0.34)	0.73 (0.34–0.93)
NPV	0.96 (0.89–0.98)	0.96 (0.90–0.98)	1.0	0.92 (0.87–0.95)
ACCURACY	0.85 (0.82–0.92)	0.90 (0.82–0.95)	0.67 (0.56–0.76)	0.92 (0.87–0.95)
ϕ	0.49	0.59	0.41	0.52
AUC	0.87	0.88	0.81	0.85

NPV, negative predictive value

**Table 5:** Variables included in the DRF model after recursive feature elimination and their importance

Variable	Relative importance	Scaled importance (0–1)
Posttreatment bicaudate index	23.29	1.00
EVD	19.87	0.85
Days with EVD	16.20	0.70
NIHSS on admission	15.01	0.64
Fisher	11.21	0.48
IVH sum score	9.76	0.42
Other Infections	7.82	0.34
IVH	5.70	0.24
WFNS	4.80	0.21
Age at SAH	4.38	0.19
mRS on admission	3.41	0.15
DCI	2.85	0.12
Aneurysm location	2.15	0.09
Hunt-Hess	1.33	0.06
KPS on admission	0.93	0.04
NIHSS motor on admission	0.67	0.03
Treatment timing from symptoms onset	0.54	0.02
Fever onset	0.47	0.02
Post-intervention ICU	0.40	0.02
ICH	0.40	0.02
ASA SCORE	0.16	0.01

Values are determined according to how much the squared error over all trees improves after the single variables is selected for splitting on a decision tree

**Fig. 1:**
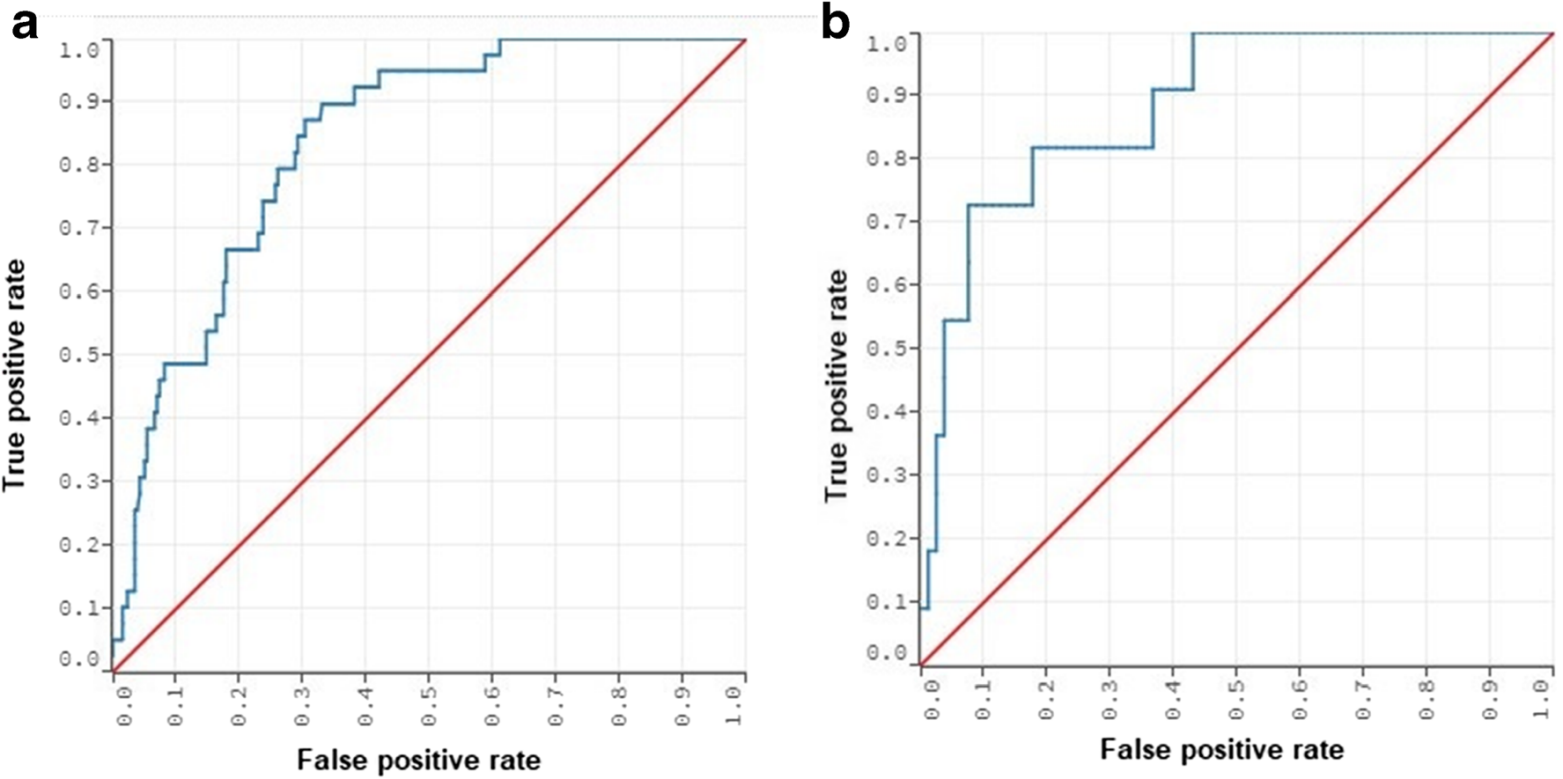
ROC curve of the model with the best performances on the **a** resampled training and **b** validation set

**Fig. 2:**
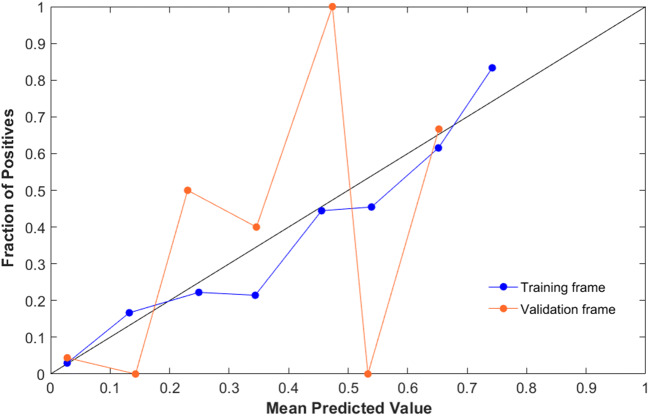
Calibration plot of the DRF model. Slope and intercept are 1.02 and 0.03 for the training frame and 0.88 and 0.07 for the validation frame

Figure 1 and Table 6 depict the AUC and the confusion matrix obtained from this model on the validation set, while Fig. 2 is a calibration plot of the model on the training and validation set: for the test set, the calibration slope and intercept were 1.02 and 0.03, respectively, whereas the calibration slope and intercept for the validation set were 0.88 and 0.07. The Hosmer-Lemeshow goodness of fit test showed a good fit of the model on both the resampled training set (*χ*^2^ = 1.7, *p* = 0.99) and the validation set (*χ*^2^ = 1.02, *p* = 1) (see also Tables 6 and 7 and Supplementary Material).

**Table 6:** Confusion matrices of the model performance on the resampled training (*n* = 296) and validation set (*n* = 90) of the model with the highest accuracy and Matthews correlation coefficient, obtained with the distributed random forest algorithm and analyzing 21 variables

Resampled training frame			
		PREDICTED	
		SDH −	SDH +
Observed	SDH −	236	21
	SDH +	20	19
Validation frame			
		PREDICTED	
		SDH −	SDH +
Observed	SDH −	73	6
	SDH +	3	8

**Table 7:** Calibration metrics from the training and validation sets

	TRAINING FRAME				VALIDATION FRAME			
Algorithm	Slope	Intercept	*χ*^2^	*p*	Slope	Intercept	*χ*^2^	*p*
GL	1.20	0.01	3.25	0.92	0.27	0.16	1.68	0.99
DRF	1.02	0.03	1.70	0.99	0.88	0.07	1.02	1.00
GBM	1.90	0.13	12.15	0.14	1.10	0.06	2.88	0.94
DL	0.57	0.14	− 29.05	1.00	0.47	0.05	− 8.22	1.00

For both sets, the slope and intercept of the calibration curve and the Hoslem-Lemeshow test *χ*2 and *p* are reported

## Discussion

In this study, we trained different machine learning models to predict the occurrence of chronic shunt-dependent hydrocephalus after aneurysmal subarachnoid hemorrhage. We further identified a single model with the best performances on previously unseen data, analyzing all variables retrieved with a distributed random forest algorithm (see Supplementary Material for details on the model parameters and the model code).

The results are comparable to previously proposed predictive models (see Table 8), which, however, took into account a limited number of variables selected after a statistical analysis of association with shunt-dependency performed with traditional methods [5, 12, 13, 20, 21, 23].

**Table 8:** Variables considered and findings of previous works proposing prognostic scores for shunt-dependent hydrocephalus

Author	Included variables	Results
Dorai et al. [13]	Hunt-Hess, sex, age, aneurysm location, IVH, clot thickness on CT	Higher scores associated with higher shunt rates
Chan et al. [5]	Hydrocephalus on admission. Hunt-Hess, CSF protein, sex, aneurysm location	Linear regression: *R*^2^ = 0.91
Jabbarli et al. 2016 [21]	Hunt-Hess, aneurysm location, hydrocephalus on admission, EVD, IVH, CIH	AUC = 0.88, association between high scores and shunt rates (*p* < 0.001)
Diesing et al. [12]	Hydrocephalus on admission, BNI, Hunt-Hess	AUC = 0.78
Hostettler et al. [20]	WFNS, hyperglycemia, aneurysm location, CRP on day 1, comorbidities, glucose on admission, leukocytes count on day 1, procalcitonin	Sensitivity and specificity on the validation set: 0.30, 0.81, respectively
Kim et al. [23]	Hydrocephalus on admission, Fisher score, age	AUC = 0.89 (95% C.I.: 0.85–0.94)

In comparison with models and scores based on previous statistical concepts, however, ML models bear the advantage of allowing more precise and subject-based predictions by including a substantial amount of variables and analyzing complex nonlinear relationships, rather than fitting the subjects’ features into predetermined models with selected and weighted variables according to statistical significance. As our experience confirms, including items that did not show a significant association with shunt-dependency when creating ML models improved the overall performances. Moreover, in the final model, variables significantly associated with shunt-dependency did not improve the overall model accuracy when used as splitting nodes in the decision trees (see also Supplementary Material). This enables to perform a more flexible and updated prediction for each subject according to small as well as relevant changes in patients’ clinical and radiological conditions.

Additionally, ML models can improve and refine autonomously when new data are provided [37], providing a dynamic model that can increase accuracy with time.

The potentials of machine learning techniques in medicine and neurosurgery have been widely tested, and their employment in diagnostic and prognostic tasks is becoming more and more common given their abilities to outperform human capacity and traditional statistics [18, 38, 41, 42, 44]. Machine learning can be considered an evolution of traditional statistics, and there is no clear line dividing them [3]. The fundamental distinction between machine learning models and traditional statistical approaches is the ability of machine learning models to independently learn from examples rather than perform a pre-programmed task [37].

Classification or regression tasks can be accomplished by supervised learning algorithms. These algorithms work with known variables (input) and outcomes (output) to detect associations between them and, once trained, can generalize this information and predict the outcome when new inputs are provided. In contrast, unsupervised learning algorithms are used to detect unknown clusters or patterns among vast amounts of data [37].

Among four of the most diffused supervised algorithms, in our experience, the most accurate model was built on a distributed random forest algorithm and included 21 items (see Table 3, Table 4 and Supplementary Material). The concept behind the DRF algorithm is to build a set of decision trees, each taking into account a subgroup of randomly selected variables and then summarizing the results of all trees either be mean or by vote to obtain an overall prediction by majority [28]. For each tree, the algorithm identifies a set of decision rules that predict the outcome based on the given variables [24]. A detailed explanation of the other tested algorithm (GL, GBM, DL) can be found here [14, 15, 26, 30, 32].

We relied on the Matthews correlation coefficient (ϕ) to identify the single best model, a metric optimized for data imbalance that is commonly used in ML and bioinformatics [4]. When the sample size in the data classes are unevenly distributed (in our case, shunt-dependent vs. non-shunt-dependent), data imbalance occurs. This frequently happens in ML, resulting in classification models maximizing the accuracy by biasing toward the majority class and leading to poor generalization [40]. In this situation, the standard measures of performance, like accuracy, are no longer a proper measure of imbalanced data. A common way to address this issue is to over-/under-sample one of the two classes [41]. However, this strategy can alter the results when the number in the minority class is limited [4].

An additional issue with model building in ML is overfitting. This is a frequent problem occurring when a too sophisticated and accurate model learns from irrelevant information or randomness of the training dataset. As a result, the predictions on new datasets will be weak. To prevent this problem, we used cross-validation, early stopping, and features removal. Also, a so-called train-test split [19] can be a sign of overfitting: when a model performs with significantly better accuracy on the training set than on the validation set, overfitting is probably occurring. In our DRF model, no train-test split differences were observed under this respect (see Tables 3 and 4).

We have to stress some limitations: first, despite all information being recalled from clinical records (i.e., prospectively acquired), the data were collected retrospectively, and we cannot exclude related biases. Second, observations of radiological scans, for example, the bicaudate index, are highly operator-dependent, and having automatized measurements would make the data more reliable. Third, some potentially relevant variables were not included, like CSF markers, or specific surgical procedures like fenestration of the lamina terminalis: CSF markers are not routinely acquired at our institution, but it would be interesting to test them in future models. Finally, despite the good metrics shown by our final model, its ability to identify patients who will actually need a permanent shunt is less accurate than its capacity to correctly exclude subjects who will not develop chronic shunt-dependent hydrocephalus. The ability to predict the development of a disease is the actual goal of any prediction model. Still, correctly ruling out future negative patients can represent a significant support for clinical decision-making and follow-up planning as well as a tool to reduce hospitalization length and costs. Additionally, it is noteworthy that the positive and negative predictive values of a test are related to the prevalence of the condition to be predicted [2]. In our cohort, the prevalence of shunt-dependency was 12.9%, and we could reasonably expect the same model to show different positive predictive values in cohorts with a different proportion of positive subjects.

## Conclusions

We trained and tested a distributed random forest model with 32 features, which reached an excellent sensitivity and specificity with ϕ = 0.59. Compared to previous models built on traditional statistical methods, it can analyze a larger amount of data and variables; work with complex nonlinear relationships; and offer a more flexible, subject-based, and accurate prognostic tool, which autonomously refines with the experience. Even though some limitations are present, prospectively testing this model performance could confirm its prognostic capacity.

## Electronic supplementary material

ESM 1 (DOCX 422 kb)

## Abbreviations

SAH: Subarachnoid hemorrhage
ML: Machine learning
CT: Computerized tomography
CTA: CT-angiography
DSA: Digital subtraction angiography
AUROC/AUC: Area under the receiver operating characteristic curve/area under the curve
PPV/NPV: Positive predictive value/negative predictive value
CI: Confidence interval
EVD: External ventricular drain
CSF: Cerebrospinal fluid
WFNS: World Federation of Neurological Surgeons
BNI: Barrow Neurological Institute
BI: Bicaudate index
GCS: Glasgow coma scale
KPS: Karnofsky performance status
ASA: American Society of Anesthesiology
mRS: Modified Rankin scale
NIHSS: National Institute of Health Stroke Scale
ICU: Intensive care unit
IMC: Intermediate care unit
GL: Generalized linear modeling
DRF: Distributed random forest
GBM: Gradient boosting machine
DL: Deep learning

## References

[1] Adams H, Ban VS, Leinonen V, Aoun SG, Huttunen J, Saavalainen T, Lindgren A, Frosen J, Fraunberg M, Koivisto T, Hernesniemi J, Welch BG, Jaaskelainen JE, Huttunen TJ (2016). Risk of shunting after aneurysmal subarachnoid hemorrhage: a collaborative study and initiation of a consortium. Stroke.

[2] Altman DG, Bland JM (1994). Statistics notes: diagnostic tests 2: predictive values. BMJ.

[3] Beam AL, Kohane IS (2018). Big data and machine learning in health care. Jama.

[4] Boughorbel S, Jarray F, El-Anbari M (2017). Optimal classifier for imbalanced data using Matthews correlation coefficient metric. PLoS One.

[5] Chan M, Alaraj A, Calderon M, Herrera SR, Gao W, Ruland S, Roitberg BZ (2009). Prediction of ventriculoperitoneal shunt dependency in patients with aneurysmal subarachnoid hemorrhage. J Neurosurg.

[6] Cleopas TJ, Zwinderman AH (2015). Machine learning in medicine.

[7] Czorlich P, Ricklefs F, Reitz M, Vettorazzi E, Abboud T, Regelsberger J, Westphal M, Schmidt NO (2015). Impact of intraventricular hemorrhage measured by Graeb and LeRoux score on case fatality risk and chronic hydrocephalus in aneurysmal subarachnoid hemorrhage. Acta Neurochir.

[8] de Oliveira JG, Beck J, Setzer M, Gerlach R, Vatter H, Seifert V, Raabe A (2007). Risk of shunt-dependent hydrocephalus after occlusion of ruptured intracranial aneurysms by surgical clipping or endovascular coiling: a single-institution series and meta-analysis. Neurosurgery.

[9] Dehdashti AR, Rilliet B, Rufenacht DA, de Tribolet N (2004). Shunt-dependent hydrocephalus after rupture of intracranial aneurysms: a prospective study of the influence of treatment modality. J Neurosurg.

[10] Deo Rahul C (2015). Machine learning in medicine. Circulation.

[11] Di Russo P, Di Carlo DT, Lutenberg A, Morganti R, Evins AI, Perrini P (2019) Shunt-dependent hydrocephalus after aneurysmal subarachnoid hemorrhage. A systematic review and meta-analysis. J Neurosurg Sci. 10.23736/s0390-5616.19.04641-1 10.23736/S0390-5616.19.04641-1 30942051

[12] Diesing D, Wolf S, Sommerfeld J, Sarrafzadeh A, Vajkoczy P, Dengler NF (2018). A novel score to predict shunt dependency after aneurysmal subarachnoid hemorrhage. J Neurosurg.

[13] Dorai Z, Hynan LS, Kopitnik TA, Samson D (2003). Factors related to hydrocephalus after aneurysmal subarachnoid hemorrhage. Neurosurgery.

[14] Friedman J, Hastie T, Tibshirani R (2010). Regularization paths for generalized linear models via coordinate descent. J Stat Softw.

[15] Friedman JH (2002). Stochastic gradient boosting. Comput Stat Data Anal.

[16] Garcia S, Torne R, Hoyos JA, Rodriguez-Hernandez A, Amaro S, Llull L, Lopez-Rueda A, Ensenat J (2018) Quantitative versus qualitative blood amount assessment as a predictor for shunt-dependent hydrocephalus following aneurysmal subarachnoid hemorrhage. J Neurosurg:1–8. 10.3171/2018.7.JNS18816 10.3171/2018.7.JNS18816 30579275

[17] Gruber A, Reinprecht A, Bavinzski G, Czech T, Richling B (1999). Chronic shunt-dependent hydrocephalus after early surgical and early endovascular treatment of ruptured intracranial aneurysms. Neurosurgery.

[18] Gulshan V, Peng L, Coram M, Stumpe MC, Wu D, Narayanaswamy A, Venugopalan S, Widner K, Madams T, Cuadros J, Kim R, Raman R, Nelson PC, Mega JL, Webster DR (2016). Development and validation of a deep learning algorithm for detection of diabetic retinopathy in retinal fundus photographs. JAMA.

[19] Hawkins DM (2004). The problem of Overfitting. J Chem Inf Comput Sci.

[20] Hostettler IC, Muroi C, Richter JK, Schmid J, Neidert MC, Seule M, Boss O, Pangalu A, Germans MR, Keller E (2018). Decision tree analysis in subarachnoid hemorrhage: prediction of outcome parameters during the course of aneurysmal subarachnoid hemorrhage using decision tree analysis. J Neurosurg.

[21] Jabbarli R, Bohrer AM, Pierscianek D, Muller D, Wrede KH, Dammann P, El Hindy N, Ozkan N, Sure U, Muller O (2016). The CHESS score: a simple tool for early prediction of shunt dependency after aneurysmal subarachnoid hemorrhage. Eur J Neurol.

[22] Jeong TS, Yoo CJ, Kim WK, Yee GT, Kim EY, Kim MJ (2018). Factors related to the development of shunt-dependent hydrocephalus following subarachnoid hemorrhage in the elderly. Turk Neurosurg.

[23] Kim JH, Kim JH, Kang HI, Kim DR, Moon BG, Kim JS (2019) Risk factors and preoperative risk scoring system for shunt-dependent hydrocephalus following aneurysmal subarachnoid hemorrhage. J Korean Neurosurg Soc. 10.3340/jkns.2018.0152 10.3340/jkns.2018.0152 PMC6835141 31064043

[24] Kuo P-J, Wu S-C, Chien P-C, Rau C-S, Chen Y-C, Hsieh H-Y, Hsieh C-H (2018). Derivation and validation of different machine-learning models in mortality prediction of trauma in motorcycle riders: a cross-sectional retrospective study in southern Taiwan. BMJ Open.

[25] Lai L, Morgan MK (2013). Predictors of in-hospital shunt-dependent hydrocephalus following rupture of cerebral aneurysms. J Clin Neurosci.

[26] LeCun Y, Bengio Y, Hinton G (2015). Deep learning. Nature.

[27] Lenski M, Biczok A, Huge V, Forbrig R, Briegel J, Tonn JC, Thon N (2019). Role of cerebrospinal fluid markers for predicting shunt-dependent hydrocephalus in patients with subarachnoid hemorrhage and external ventricular drain placement. World Neurosurg.

[28] Li J, Tian Y, Zhu Y, Zhou T, Li J, Ding K, Li J (2020). A multicenter random forest model for effective prognosis prediction in collaborative clinical research network. Artif Intell Med.

[29] Mijderwijk HJ, Fischer I, Zhivotovskaya A, Bostelmann R, Steiger HJ, Cornelius JF, Petridis AK (2019) Prognostic model for chronic shunt-dependent hydrocephalus after aneurysmal subarachnoid hemorrhage. World Neurosurg. 10.1016/j.wneu.2018.12.156 10.1016/j.wneu.2018.12.156 30639492

[30] Miotto R, Wang F, Wang S, Jiang X, Dudley JT (2017). Deep learning for healthcare: review, opportunities and challenges. Brief Bioinform.

[31] Na MK, Won YD, Kim CH, Kim JM, Cheong JH, Ryu JI, Han MH (2017). Early variations of laboratory parameters predicting shunt-dependent hydrocephalus after subarachnoid hemorrhage. PLoS One.

[32] Natekin A, Knoll A (2013). Gradient boosting machines, a tutorial. Front Neurorobot.

[33] O'Kelly CJ, Kulkarni AV, Austin PC, Urbach D, Wallace MC (2009). Shunt-dependent hydrocephalus after aneurysmal subarachnoid hemorrhage: incidence, predictors, and revision rates. J Neurosurg.

[34] Obermeyer Z, Emanuel EJ (2016). Predicting the future - big data, machine learning, and clinical medicine. N Engl J Med.

[35] Paisan GM, Ding D, Starke RM, Crowley RW, Liu KC (2018). Shunt-dependent hydrocephalus after aneurysmal subarachnoid hemorrhage: predictors and long-term functional outcomes. Neurosurgery.

[36] Park YK, Yi HJ, Choi KS, Lee YJ, Chun HJ, Kwon SM, Kim DW (2018). Predicting factors for shunt-dependent hydrocephalus in patients with aneurysmal subarachnoid hemorrhage. Acta Neurochir.

[37] Rajkomar A, Dean J, Kohane I (2019). Machine learning in medicine. N Engl J Med.

[38] Ramos LA, van der Steen WE, Sales Barros R, Majoie C, van den Berg R, Verbaan D, Vandertop WP, Zijlstra I, Zwinderman AH, Strijkers GJ, Olabarriaga SD, Marquering HA (2019). Machine learning improves prediction of delayed cerebral ischemia in patients with subarachnoid hemorrhage. J Neurointerv Surg.

[39] Rincon F, Gordon E, Starke RM, Buitrago MM, Fernandez A, Schmidt JM, Claassen J, Wartenberg KE, Frontera J, Seder DB, Palestrant D, Connolly ES, Lee K, Mayer SA, Badjatia N (2010). Predictors of long-term shunt-dependent hydrocephalus after aneurysmal subarachnoid hemorrhage. Clinical article. J Neurosurg.

[40] Staartjes VE, Schroder ML (2018). Letter to the editor. Class imbalance in machine learning for neurosurgical outcome prediction: are our models valid?. J Neurosurg Spine.

[41] Staartjes VE, Serra C, Muscas G, Maldaner N, Akeret K, van Niftrik CHB, Fierstra J, Holzmann D, Regli L (2018). Utility of deep neural networks in predicting gross-total resection after transsphenoidal surgery for pituitary adenoma: a pilot study. Neurosurg Focus.

[42] Staartjes VE, Zattra CM, Akeret K, Maldaner N, Muscas G, Bas van Niftrik CH, Fierstra J, Regli L, Serra C (2019) Neural network-based identification of patients at high risk for intraoperative cerebrospinal fluid leaks in endoscopic pituitary surgery. J Neurosurg:1–7. 10.3171/2019.4.Jns19477 10.3171/2019.4.JNS19477 31226693

[43] Tso MK, Ibrahim GM, Macdonald RL (2016). Predictors of shunt-dependent hydrocephalus following aneurysmal subarachnoid hemorrhage. World Neurosurg.

[44] van Niftrik CHB, van der Wouden F, Staartjes VE, Fierstra J, Stienen MN, Akeret K, Sebok M, Fedele T, Sarnthein J, Bozinov O, Krayenbuhl N, Regli L, Serra C (2019) Machine learning algorithm identifies patients at high risk for early complications after intracranial tumor surgery: registry-based cohort study. Neurosurgery. 10.1093/neuros/nyz145 10.1093/neuros/nyz145 31149726

[45] Wilson CD, Safavi-Abbasi S, Sun H, Kalani MYS, Zhao YD, Levitt MR, Hanel RA, Sauvageau E, Mapstone TB, Albuquerque FC, McDougall CG, Nakaji P, Spetzler RF (2017) Meta-analysis and systematic review of risk factors for shunt dependency after aneurysmal subarachnoid hemorrhage. 126:586. 10.3171/2015.11.Jns152094 10.3171/2015.11.JNS152094 27035169

[46] Wostrack M, Reeb T, Martin J, Kehl V, Shiban E, Preuss A, Ringel F, Meyer B, Ryang YM (2014). Shunt-dependent hydrocephalus after aneurysmal subarachnoid hemorrhage: the role of intrathecal interleukin-6. Neurocrit Care.

[47] Xie Z, Hu X, Zan X, Lin S, Li H, You C (2017). Predictors of shunt-dependent hydrocephalus after aneurysmal subarachnoid hemorrhage? A Systematic Review and Meta-Analysis. World Neurosurg.

[48] Yang T-C, Chang CH, Liu Y-T, Chen Y-L, Tu P-H, Chen H-C (2013). Predictors of shunt-dependent chronic hydrocephalus after aneurysmal subarachnoid haemorrhage. Eur Neurol.

[49] Zaidi HA, Montoure A, Elhadi A, Nakaji P, McDougall CG, Albuquerque FC, Spetzler RF, Zabramski JM (2015). Long-term functional outcomes and predictors of shunt-dependent hydrocephalus after treatment of ruptured intracranial aneurysms in the BRAT trial: revisiting the clip vs coil debate. Neurosurgery.

[50] Zeng J, Qin L, Wang D, Gong J, Pan J, Zhu Y, Sun T, Xu K, Zhan R (2019). Comparing the risk of shunt-dependent hydrocephalus in patients with ruptured intracranial aneurysms treated by endovascular coiling or surgical clipping: an updated meta-analysis. World Neurosurg.

