# Supplementary material for: Development of machine learning models to prognosticate chronic shunt-dependent hydrocephalus after aneurysmal subarachnoid hemorrhage
Source: Acta Neurochir (Wien). 2020 Jul 8;162(12):3093–105. doi: 10.1007/s00701-020-04484-6 (PMC7593274; doi:10.1007/s00701-020-04484-6)
Supplement: Supplementary file 1 — (DOCX 422 kb) [file 701_2020_4484_MOESM1_ESM.docx]

**SUPPLEMENTARY MATERIAL**

1. **Confusion matrices, ROC curves and calibration plots of the models obtained from the resampled training and validation sets.** Please refer to the main manuscript for the Distributed Random Forest model
   1. **GENERALIZED LINEAR MODEL**

**Variables included:** Days with EVD, Fisher, EVD (Yes/No), Post-operative bicaudate Index, Infections, Sex, days with Fever, IVH-sum score, age, treatment modality, ICH


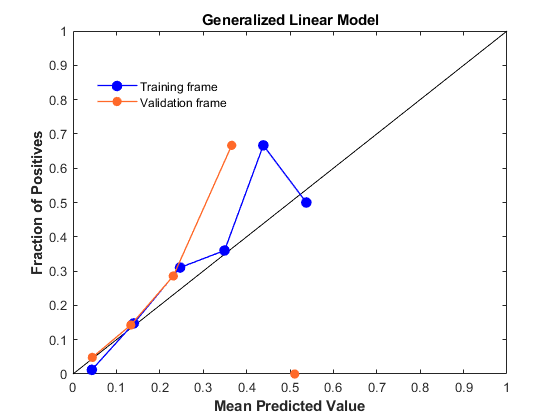


**Resampled training frame**

|  |  | PREDICTED | |
| --- | --- | --- | --- |
|  |  | SDH - | SDH + |
| OBSERVED | SDH - | 204 | 53 |
|  | SDH + | 9 | 30 |

**Validation frame**

|  |  | PREDICTED | |
| --- | --- | --- | --- |
|  |  | SDH - | SDH + |
| OBSERVED | SDH - | 69 | 10 |
|  | SDH + | 3 | 8 |


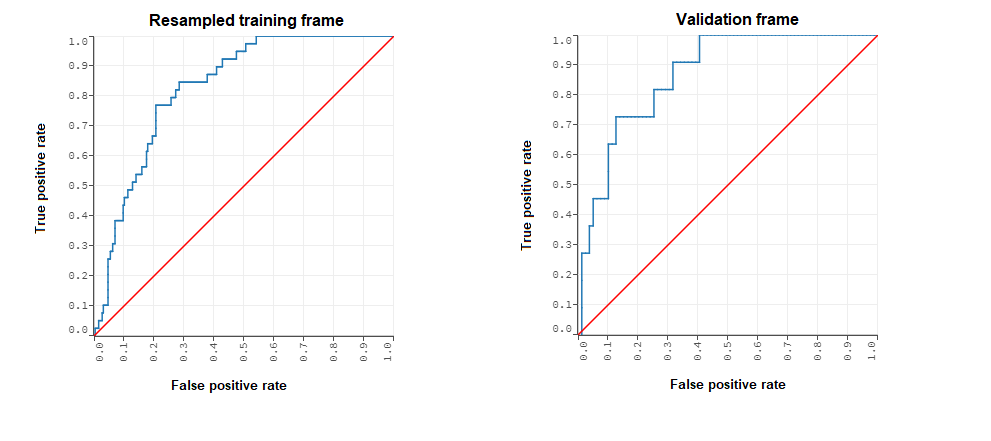


**4.2. GRADIENT BOOSTING MACHINE**

**Variables included:** Days with EVD, Post-treatment Bicaudate Index, Aneurysm location, Age, IVH-sum score, Aneurysm diameter, Treatment timing, Multiple Aneurysms (Yes/No), Treatment modality, Sex, ICH, Acute hydrocephalus, Infections, Hunt-Hess, NIHSS on admission, mRS on admission, KPS on admission, Treatment complication, ASA SCORE, DCI, Rehemorrage, WFNS, NIHSS Motor , Meningitis, IVH, Fisher, Days with fever, Post-intervention ICU

**
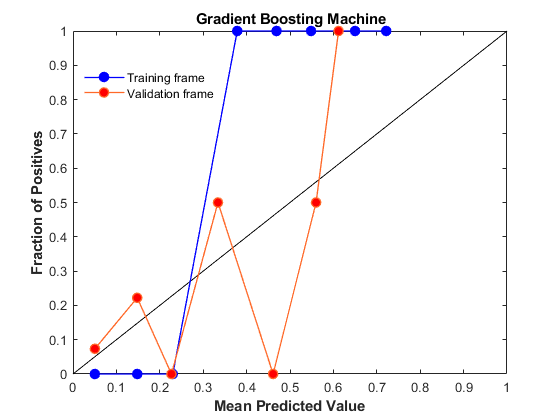
Resampled training frame**

|  |  | PREDICTED | |
| --- | --- | --- | --- |
|  |  | SDH - | SDH + |
| OBSERVED | SDH - | 220 | 37 |
|  | SDH + | 16 | 23 |

**Validation frame**

|  |  | PREDICTED | |
| --- | --- | --- | --- |
|  |  | SDH - | SDH + |
| OBSERVED | SDH - | 49 | 30 |
|  | SDH + | 0 | 11 |

**
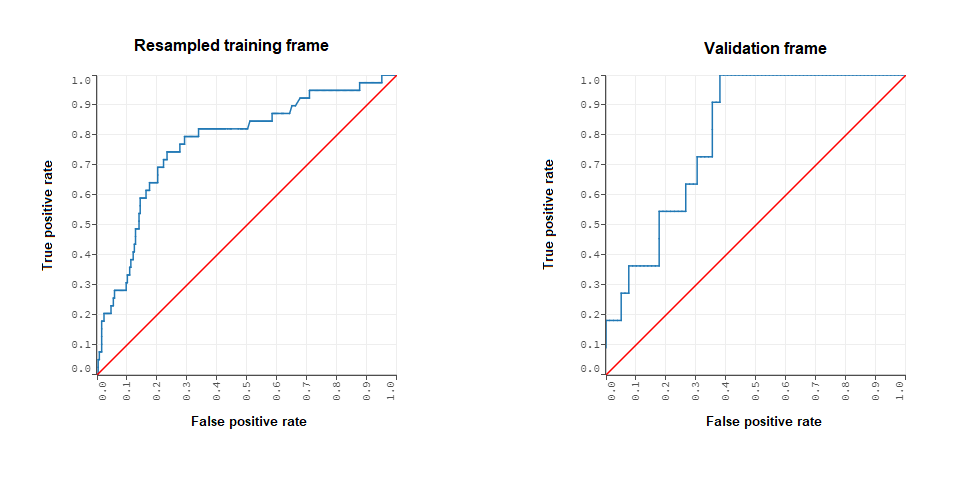
**

**4.3. DEEP LEARNING**

**Variables included:** all

**
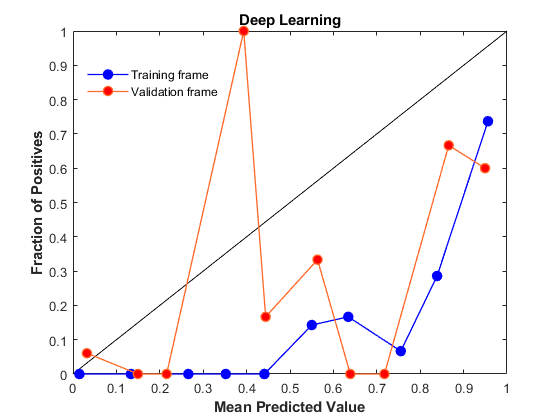
**

**Resampled training frame**

|  |  | PREDICTED | |
| --- | --- | --- | --- |
|  |  | SDH - | SDH + |
| OBSERVED | SDH - | 205 | 52 |
|  | SDH + | 11 | 28 |

**Validation frame**

|  |  | PREDICTED | |
| --- | --- | --- | --- |
|  |  | SDH - | SDH + |
| OBSERVED | SDH - | 77 | 2 |
|  | SDH + | 6 | 5 |


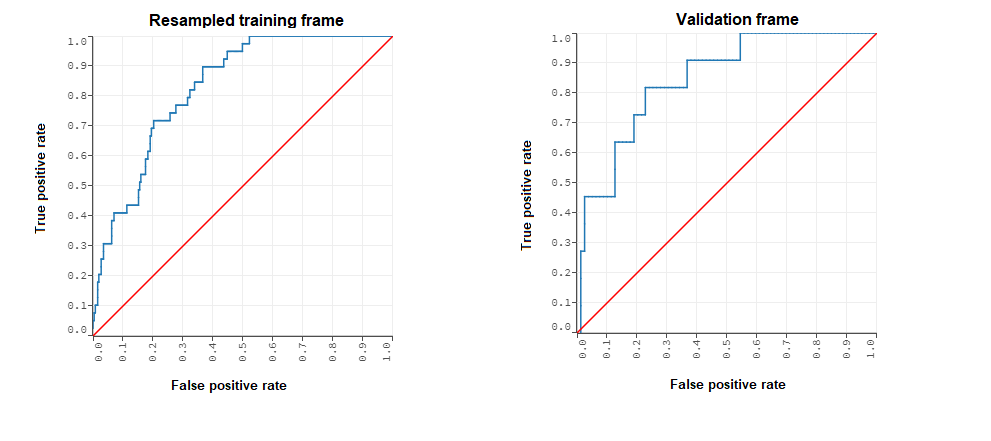


1. **Code of the Distributed Random Forest model (H2O Flow, www.h2o.ai, Mountain View, CA, USA)**

**How to download, compile and execute:**

**mkdir tmpdir**

**cd tmpdir**

**curl http://172.18.11.118:54321/3/h2o-genmodel.jar > h2o-genmodel.jar**

**curl http://172.18.11.118:54321/3/Models.java/DRF-4 > DRF_4.java**

**javac -cp h2o-genmodel.jar -J-Xmx2g -J-XX:MaxPermSize=128m DRF_4.java**

**(Note: Try java argument -XX:+PrintCompilation to show runtime JIT compiler behavior.)**

***/**

**import java.util.Map;**

**import hex.genmodel.GenModel;**

**import hex.genmodel.annotations.ModelPojo;**

@ModelPojo(name="DRF_4", algorithm="drf")

public class DRF_4 extends GenModel {

public hex.ModelCategory getModelCategory() { return hex.ModelCategory.Binomial; }

public boolean isSupervised() { return true; }

public int nfeatures() { return 32; }

public int nclasses() { return 2; }

// Names of columns used by model.

public static final String[] NAMES = NamesHolder_DRF_4.VALUES;

// Number of output classes included in training data response column.

public static final int NCLASSES = 2;

// Column domains. The last array contains domain of response column.

public static final String[][] DOMAINS = new String[][] {

/* Age at SAH */ null,

/* Sex */ DRF_4_ColInfo_1.VALUES,

/* GCS at admission */ DRF_4_ColInfo_2.VALUES,

/* Fisher */ null,

/* Hunt-Hess */ null,

/* WFNS */ null,

/* ICH */ DRF_4_ColInfo_6.VALUES,

/* IVH */ DRF_4_ColInfo_7.VALUES,

/* Treatment timing from symptoms onset */ DRF_4_ColInfo_8.VALUES,

/* IVH sum score */ null,

/* Post-treatment Bicaudate Index */ null,

/* mRS on admission */ null,

/* ASA SCORE */ null,

/* KPS on admission */ null,

/* NIHSS on admission */ null,

/* NIHSS Motor on admission */ null,

/* Initial acute hydrocephalus */ DRF_4_ColInfo_16.VALUES,

/* EVD */ DRF_4_ColInfo_17.VALUES,

/* Days with EVD */ DRF_4_ColInfo_18.VALUES,

/* Rehemorrage */ DRF_4_ColInfo_19.VALUES,

/* Aneurysm location */ DRF_4_ColInfo_20.VALUES,

/* Multiple Aneurysms */ DRF_4_ColInfo_21.VALUES,

/* MAX Aneurysm diameter (mm) */ null,

/* Treatment modality */ DRF_4_ColInfo_23.VALUES,

/* Post-intervention ICU */ DRF_4_ColInfo_24.VALUES,

/* DCI */ DRF_4_ColInfo_25.VALUES,

/* Treatment complication */ DRF_4_ColInfo_26.VALUES,

/* Fever */ DRF_4_ColInfo_27.VALUES,

/* Fever onset */ DRF_4_ColInfo_28.VALUES,

/* Days with fever */ DRF_4_ColInfo_29.VALUES,

/* Meningitis */ DRF_4_ColInfo_30.VALUES,

/* Other Infections */ DRF_4_ColInfo_31.VALUES,

/* VPS */ DRF_4_ColInfo_32.VALUES

};

// Prior class distribution

public static final double[] PRIOR_CLASS_DISTRIB = {0.8682432432432432,0.13175675675675674};

// Class distribution used for model building

public static final double[] MODEL_CLASS_DISTRIB = {0.8682432432432432,0.13175675675675674};

public DRF_4() { super(NAMES,DOMAINS,"VPS"); }

public String getUUID() { return Long.toString(4335061002119740864L); }

// Pass in data in a double[], pre-aligned to the Model's requirements.

// Jam predictions into the preds[] array; preds[0] is reserved for the

// main prediction (class for classifiers or value for regression),

// and remaining columns hold a probability distribution for classifiers.

public final double[] score0( double[] data, double[] preds ) {

java.util.Arrays.fill(preds,0);

DRF_4_Forest_0.score0(data,preds);

DRF_4_Forest_1.score0(data,preds);

DRF_4_Forest_2.score0(data,preds);

DRF_4_Forest_3.score0(data,preds);

DRF_4_Forest_4.score0(data,preds);

DRF_4_Forest_5.score0(data,preds);

DRF_4_Forest_6.score0(data,preds);

DRF_4_Forest_7.score0(data,preds);

DRF_4_Forest_8.score0(data,preds);

DRF_4_Forest_9.score0(data,preds);

DRF_4_Forest_10.score0(data,preds);

DRF_4_Forest_11.score0(data,preds);

double sum = 0;

for(int i=1; i<preds.length; i++) { sum += preds[i]; }

if (sum>0) for(int i=1; i<preds.length; i++) { preds[i] /= sum; }

preds[0] = hex.genmodel.GenModel.getPrediction(preds, PRIOR_CLASS_DISTRIB, data, 0.23660708385135057);

return preds;

}

}

// The class representing training column names

class NamesHolder_DRF_4 implements java.io.Serializable {

public static final String[] VALUES = new String[32];

static {

NamesHolder_DRF_4_0.fill(VALUES);

}

static final class NamesHolder_DRF_4_0 implements java.io.Serializable {

static final void fill(String[] sa) {

sa[0] = "Age at SAH";

sa[1] = "Sex";

sa[2] = "GCS at admission";

sa[3] = "Fisher";

sa[4] = "Hunt-Hess";

sa[5] = "WFNS";

sa[6] = "ICH";

sa[7] = "IVH";

sa[8] = "Treatment timing from symptoms onset";

sa[9] = "IVH sum score";

sa[10] = "Post-treatment Bicaudate Index";

sa[11] = "mRS on admission";

sa[12] = "ASA SCORE";

sa[13] = "KPS on admission";

sa[14] = "NIHSS on admission";

sa[15] = "NIHSS Motor on admission";

sa[16] = "Initial acute hydrocephalus";

sa[17] = "EVD";

sa[18] = "Days with EVD";

sa[19] = "Rehemorrage";

sa[20] = "Aneurysm location";

sa[21] = "Multiple Aneurysms";

sa[22] = "MAX Aneurysm diameter (mm)";

sa[23] = "Treatment modality";

sa[24] = "Post-intervention ICU";

sa[25] = "DCI";

sa[26] = "Treatment complication";

sa[27] = "Fever";

sa[28] = "Fever onset";

sa[29] = "Days with fever";

sa[30] = "Meningitis";

sa[31] = "Other Infections";

}

}

}

// The class representing column Sex

class DRF_4_ColInfo_1 implements java.io.Serializable {

public static final String[] VALUES = new String[2];

static {

DRF_4_ColInfo_1_0.fill(VALUES);

}

static final class DRF_4_ColInfo_1_0 implements java.io.Serializable {

static final void fill(String[] sa) {

sa[0] = "F";

sa[1] = "M";

}

}

}

// The class representing column GCS at admission

class DRF_4_ColInfo_2 implements java.io.Serializable {

public static final String[] VALUES = new String[3];

static {

DRF_4_ColInfo_2_0.fill(VALUES);

}

static final class DRF_4_ColInfo_2_0 implements java.io.Serializable {

static final void fill(String[] sa) {

sa[0] = "12 - 15";

sa[1] = "8 - 20";

sa[2] = "<8";

}

}

}

// The class representing column ICH

class DRF_4_ColInfo_6 implements java.io.Serializable {

public static final String[] VALUES = new String[2];

static {

DRF_4_ColInfo_6_0.fill(VALUES);

}

static final class DRF_4_ColInfo_6_0 implements java.io.Serializable {

static final void fill(String[] sa) {

sa[0] = "No";

sa[1] = "Yes";

}

}

}

// The class representing column IVH

class DRF_4_ColInfo_7 implements java.io.Serializable {

public static final String[] VALUES = new String[2];

static {

DRF_4_ColInfo_7_0.fill(VALUES);

}

static final class DRF_4_ColInfo_7_0 implements java.io.Serializable {

static final void fill(String[] sa) {

sa[0] = "No";

sa[1] = "Yes";

}

}

}

// The class representing column Treatment timing from symptoms onset

class DRF_4_ColInfo_8 implements java.io.Serializable {

public static final String[] VALUES = new String[4];

static {

DRF_4_ColInfo_8_0.fill(VALUES);

}

static final class DRF_4_ColInfo_8_0 implements java.io.Serializable {

static final void fill(String[] sa) {

sa[0] = "12-24 hours";

sa[1] = "6-12 hours";

sa[2] = "<6 hours";

sa[3] = ">24 hours";

}

}

}

// The class representing column Initial acute hydrocephalus

class DRF_4_ColInfo_16 implements java.io.Serializable {

public static final String[] VALUES = new String[2];

static {

DRF_4_ColInfo_16_0.fill(VALUES);

}

static final class DRF_4_ColInfo_16_0 implements java.io.Serializable {

static final void fill(String[] sa) {

sa[0] = "No";

sa[1] = "Yes";

}

}

}

// The class representing column EVD

class DRF_4_ColInfo_17 implements java.io.Serializable {

public static final String[] VALUES = new String[2];

static {

DRF_4_ColInfo_17_0.fill(VALUES);

}

static final class DRF_4_ColInfo_17_0 implements java.io.Serializable {

static final void fill(String[] sa) {

sa[0] = "No";

sa[1] = "Yes";

}

}

}

// The class representing column Days with EVD

class DRF_4_ColInfo_18 implements java.io.Serializable {

public static final String[] VALUES = new String[2];

static {

DRF_4_ColInfo_18_0.fill(VALUES);

}

static final class DRF_4_ColInfo_18_0 implements java.io.Serializable {

static final void fill(String[] sa) {

sa[0] = "<=5";

sa[1] = ">5";

}

}

}

// The class representing column Rehemorrage

class DRF_4_ColInfo_19 implements java.io.Serializable {

public static final String[] VALUES = new String[2];

static {

DRF_4_ColInfo_19_0.fill(VALUES);

}

static final class DRF_4_ColInfo_19_0 implements java.io.Serializable {

static final void fill(String[] sa) {

sa[0] = "No";

sa[1] = "Yes";

}

}

}

// The class representing column Aneurysm location

class DRF_4_ColInfo_20 implements java.io.Serializable {

public static final String[] VALUES = new String[13];

static {

DRF_4_ColInfo_20_0.fill(VALUES);

}

static final class DRF_4_ColInfo_20_0 implements java.io.Serializable {

static final void fill(String[] sa) {

sa[0] = "ACA";

sa[1] = "AICA";

sa[2] = "Ach";

sa[3] = "AcoA";

sa[4] = "B";

sa[5] = "CS";

sa[6] = "MCA";

sa[7] = "O";

sa[8] = "PCA";

sa[9] = "PICA";

sa[10] = "Pcom";

sa[11] = "Pericallosal";

sa[12] = "V";

}

}

}

// The class representing column Multiple Aneurysms

class DRF_4_ColInfo_21 implements java.io.Serializable {

public static final String[] VALUES = new String[7];

static {

DRF_4_ColInfo_21_0.fill(VALUES);

}

static final class DRF_4_ColInfo_21_0 implements java.io.Serializable {

static final void fill(String[] sa) {

sa[0] = "No";

sa[1] = "No,23";

sa[2] = "No,28";

sa[3] = "No,3";

sa[4] = "No,Yes2";

sa[5] = "No,Yes4";

sa[6] = "Yes";

}

}

}

// The class representing column Treatment modality

class DRF_4_ColInfo_23 implements java.io.Serializable {

public static final String[] VALUES = new String[2];

static {

DRF_4_ColInfo_23_0.fill(VALUES);

}

static final class DRF_4_ColInfo_23_0 implements java.io.Serializable {

static final void fill(String[] sa) {

sa[0] = "Endovascular";

sa[1] = "Surgical";

}

}

}

// The class representing column Post-intervention ICU

class DRF_4_ColInfo_24 implements java.io.Serializable {

public static final String[] VALUES = new String[2];

static {

DRF_4_ColInfo_24_0.fill(VALUES);

}

static final class DRF_4_ColInfo_24_0 implements java.io.Serializable {

static final void fill(String[] sa) {

sa[0] = "No";

sa[1] = "Yes";

}

}

}

// The class representing column DCI

class DRF_4_ColInfo_25 implements java.io.Serializable {

public static final String[] VALUES = new String[4];

static {

DRF_4_ColInfo_25_0.fill(VALUES);

}

static final class DRF_4_ColInfo_25_0 implements java.io.Serializable {

static final void fill(String[] sa) {

sa[0] = "No";

sa[1] = "No,37";

sa[2] = "No,Yes2";

sa[3] = "Yes";

}

}

}

// The class representing column Treatment complication

class DRF_4_ColInfo_26 implements java.io.Serializable {

public static final String[] VALUES = new String[3];

static {

DRF_4_ColInfo_26_0.fill(VALUES);

}

static final class DRF_4_ColInfo_26_0 implements java.io.Serializable {

static final void fill(String[] sa) {

sa[0] = "No";

sa[1] = "No,28";

sa[2] = "Yes";

}

}

}

// The class representing column Fever

class DRF_4_ColInfo_27 implements java.io.Serializable {

public static final String[] VALUES = new String[2];

static {

DRF_4_ColInfo_27_0.fill(VALUES);

}

static final class DRF_4_ColInfo_27_0 implements java.io.Serializable {

static final void fill(String[] sa) {

sa[0] = "No";

sa[1] = "Yes";

}

}

}

// The class representing column Fever onset

class DRF_4_ColInfo_28 implements java.io.Serializable {

public static final String[] VALUES = new String[3];

static {

DRF_4_ColInfo_28_0.fill(VALUES);

}

static final class DRF_4_ColInfo_28_0 implements java.io.Serializable {

static final void fill(String[] sa) {

sa[0] = "Delayed";

sa[1] = "Early (<7 days)";

sa[2] = "No fever";

}

}

}

// The class representing column Days with fever

class DRF_4_ColInfo_29 implements java.io.Serializable {

public static final String[] VALUES = new String[2];

static {

DRF_4_ColInfo_29_0.fill(VALUES);

}

static final class DRF_4_ColInfo_29_0 implements java.io.Serializable {

static final void fill(String[] sa) {

sa[0] = "<=5";

sa[1] = ">5";

}

}

}

// The class representing column Meningitis

class DRF_4_ColInfo_30 implements java.io.Serializable {

public static final String[] VALUES = new String[6];

static {

DRF_4_ColInfo_30_0.fill(VALUES);

}

static final class DRF_4_ColInfo_30_0 implements java.io.Serializable {

static final void fill(String[] sa) {

sa[0] = "No";

sa[1] = "No,25";

sa[2] = "No,26";

sa[3] = "No,Yes2";

sa[4] = "No,Yes5";

sa[5] = "Yes";

}

}

}

// The class representing column Other Infections

class DRF_4_ColInfo_31 implements java.io.Serializable {

public static final String[] VALUES = new String[2];

static {

DRF_4_ColInfo_31_0.fill(VALUES);

}

static final class DRF_4_ColInfo_31_0 implements java.io.Serializable {

static final void fill(String[] sa) {

sa[0] = "No";

sa[1] = "Yes";

}

}

}

// The class representing column VPS

class DRF_4_ColInfo_32 implements java.io.Serializable {

public static final String[] VALUES = new String[2];

static {

DRF_4_ColInfo_32_0.fill(VALUES);

}

static final class DRF_4_ColInfo_32_0 implements java.io.Serializable {

static final void fill(String[] sa) {

sa[0] = "No";

sa[1] = "Yes";

}

}

}

class DRF_4_Forest_0 {

public static void score0(double[] fdata, double[] preds) {

preds[1] += DRF_4_Tree_0_class_0.score0(fdata);

preds[2] += DRF_4_Tree_0_class_1.score0(fdata);

}

}

class DRF_4_Tree_0_class_0 {

static final double score0(double[] data) {

double pred = (!Double.isNaN(data[18 /* Days with EVD */]) && (GenModel.bitSetIsInRange(32, 0, data[18]) && !GenModel.bitSetContains(GRPSPLIT0, 32, 0, data[18])) ?

(data[11 /* mRS on admission */] <2.5f ?

0.8095238f :

0.6315789f) :

(!Double.isNaN(data[8 /* Treatment timing from symptoms onset */]) && (GenModel.bitSetIsInRange(32, 0, data[8]) && !GenModel.bitSetContains(GRPSPLIT1, 32, 0, data[8])) ?

(!Double.isNaN(data[7 /* IVH */]) && (GenModel.bitSetIsInRange(32, 0, data[7]) && !GenModel.bitSetContains(GRPSPLIT2, 32, 0, data[7])) ?

0.89285713f :

0.96875f) :

(!Double.isNaN(data[7 /* IVH */]) && (GenModel.bitSetIsInRange(32, 0, data[7]) && !GenModel.bitSetContains(GRPSPLIT3, 32, 0, data[7])) ?

0.95238096f :

1.0f)));

return pred;

} // constant pool size = 42B, number of visited nodes = 5, static init size = 120B

// {10000000 00000000 00000000 00000000}

public static final byte[] GRPSPLIT0 = new byte[] {1, 0, 0, 0};

// {10010000 00000000 00000000 00000000}

public static final byte[] GRPSPLIT1 = new byte[] {9, 0, 0, 0};

// {10000000 00000000 00000000 00000000}

public static final byte[] GRPSPLIT2 = new byte[] {1, 0, 0, 0};

// {10000000 00000000 00000000 00000000}

public static final byte[] GRPSPLIT3 = new byte[] {1, 0, 0, 0};

}

class DRF_4_Tree_0_class_1 {

static final double score0(double[] data) {

double pred = (Double.isNaN(data[18 /* Days with EVD */]) || !GenModel.bitSetIsInRange(32, 0, data[18]) || (GenModel.bitSetIsInRange(32, 0, data[18]) && !GenModel.bitSetContains(GRPSPLIT0, 32, 0, data[18])) ?

(Double.isNaN(data[31 /* Other Infections */]) || !GenModel.bitSetIsInRange(32, 0, data[31]) || (GenModel.bitSetIsInRange(32, 0, data[31]) && !GenModel.bitSetContains(GRPSPLIT1, 32, 0, data[31])) ?

0.0f :

(Double.isNaN(data[9]) || data[9 /* IVH sum score */] <1.5f ?

0.04761905f :

0.2f)) :

(Double.isNaN(data[12]) || data[12 /* ASA SCORE */] <2.5f ?

0.2820513f :

0.35f));

return pred;

} // constant pool size = 28B, number of visited nodes = 4, static init size = 60B

// {01000000 00000000 00000000 00000000}

public static final byte[] GRPSPLIT0 = new byte[] {2, 0, 0, 0};

// {01000000 00000000 00000000 00000000}

public static final byte[] GRPSPLIT1 = new byte[] {2, 0, 0, 0};

}

class DRF_4_Forest_1 {

public static void score0(double[] fdata, double[] preds) {

preds[1] += DRF_4_Tree_1_class_0.score0(fdata);

preds[2] += DRF_4_Tree_1_class_1.score0(fdata);

}

}

class DRF_4_Tree_1_class_0 {

static final double score0(double[] data) {

double pred = (Double.isNaN(data[5]) || data[5 /* WFNS */] <1.5f ?

(Double.isNaN(data[10]) || data[10 /* Post-treatment Bicaudate Index */] <0.175f ?

(Double.isNaN(data[3]) || data[3 /* Fisher */] <3.5f ?

1.0f :

0.90909094f) :

0.85714287f) :

(Double.isNaN(data[0]) || data[0 /* Age at SAH */] <63.5f ?

(Double.isNaN(data[14]) || data[14 /* NIHSS on admission */] <22.5f ?

0.8055556f :

0.95f) :

0.6f));

return pred;

} // constant pool size = 22B, number of visited nodes = 5, static init size = 0B

}

class DRF_4_Tree_1_class_1 {

static final double score0(double[] data) {

double pred = (Double.isNaN(data[5]) || data[5 /* WFNS */] <1.5f ?

(Double.isNaN(data[10]) || data[10 /* Post-treatment Bicaudate Index */] <0.175f ?

(Double.isNaN(data[3]) || data[3 /* Fisher */] <3.5f ?

0.0f :

0.09090909f) :

0.14285715f) :

(Double.isNaN(data[0]) || data[0 /* Age at SAH */] <63.5f ?

(Double.isNaN(data[14]) || data[14 /* NIHSS on admission */] <22.5f ?

0.19444445f :

0.05f) :

0.4f));

return pred;

} // constant pool size = 22B, number of visited nodes = 5, static init size = 0B

}

class DRF_4_Forest_2 {

public static void score0(double[] fdata, double[] preds) {

preds[1] += DRF_4_Tree_2_class_0.score0(fdata);

preds[2] += DRF_4_Tree_2_class_1.score0(fdata);

}

}

class DRF_4_Tree_2_class_0 {

static final double score0(double[] data) {

double pred = (!Double.isNaN(data[17 /* EVD */]) && (GenModel.bitSetIsInRange(32, 0, data[17]) && !GenModel.bitSetContains(GRPSPLIT0, 32, 0, data[17])) ?

(Double.isNaN(data[13]) || data[13 /* KPS on admission */] <54.5f ?

0.5483871f :

0.7692308f) :

(Double.isNaN(data[20 /* Aneurysm location */]) || !GenModel.bitSetIsInRange(32, 0, data[20]) || (GenModel.bitSetIsInRange(32, 0, data[20]) && !GenModel.bitSetContains(GRPSPLIT1, 32, 0, data[20])) ?

(!Double.isNaN(data[31 /* Other Infections */]) && (GenModel.bitSetIsInRange(32, 0, data[31]) && !GenModel.bitSetContains(GRPSPLIT2, 32, 0, data[31])) ?

0.8484849f :

(data[4 /* Hunt-Hess */] <1.5f ?

1.0f :

0.9459459f)) :

1.0f));

return pred;

} // constant pool size = 37B, number of visited nodes = 5, static init size = 90B

// {10000000 00000000 00000000 00000000}

public static final byte[] GRPSPLIT0 = new byte[] {1, 0, 0, 0};

// {01101101 11111000 00000000 00000000}

public static final byte[] GRPSPLIT1 = new byte[] {-74, 31, 0, 0};

// {10000000 00000000 00000000 00000000}

public static final byte[] GRPSPLIT2 = new byte[] {1, 0, 0, 0};

}

class DRF_4_Tree_2_class_1 {

static final double score0(double[] data) {

double pred = (Double.isNaN(data[17 /* EVD */]) || !GenModel.bitSetIsInRange(32, 0, data[17]) || (GenModel.bitSetIsInRange(32, 0, data[17]) && !GenModel.bitSetContains(GRPSPLIT0, 32, 0, data[17])) ?

(Double.isNaN(data[4]) || data[4 /* Hunt-Hess */] <2.5f ?

(data[10 /* Post-treatment Bicaudate Index */] <0.11484375f ?

0.0f :

(data[13 /* KPS on admission */] <75.0f ?

0.05f :

0.028571429f)) :

(data[13 /* KPS on admission */] <36.0f ?

0.05f :

0.14285715f)) :

(!Double.isNaN(data[25 /* DCI */]) && (GenModel.bitSetIsInRange(32, 0, data[25]) && !GenModel.bitSetContains(GRPSPLIT1, 32, 0, data[25])) ?

0.23076923f :

0.4516129f));

return pred;

} // constant pool size = 36B, number of visited nodes = 6, static init size = 60B

// {01000000 00000000 00000000 00000000}

public static final byte[] GRPSPLIT0 = new byte[] {2, 0, 0, 0};

// {00010000 00000000 00000000 00000000}

public static final byte[] GRPSPLIT1 = new byte[] {8, 0, 0, 0};

}

class DRF_4_Forest_3 {

public static void score0(double[] fdata, double[] preds) {

preds[1] += DRF_4_Tree_3_class_0.score0(fdata);

preds[2] += DRF_4_Tree_3_class_1.score0(fdata);

}

}

class DRF_4_Tree_3_class_0 {

static final double score0(double[] data) {

double pred = (data[3 /* Fisher */] <3.5f ?

(Double.isNaN(data[10]) || data[10 /* Post-treatment Bicaudate Index */] <0.175f ?

(data[12 /* ASA SCORE */] <1.5f ?

1.0f :

(Double.isNaN(data[10]) || data[10 /* Post-treatment Bicaudate Index */] <0.13535155f ?

1.0f :

0.95238096f)) :

0.8636364f) :

(!Double.isNaN(data[25 /* DCI */]) && (GenModel.bitSetIsInRange(32, 0, data[25]) && !GenModel.bitSetContains(GRPSPLIT0, 32, 0, data[25])) ?

0.65f :

(Double.isNaN(data[9]) || data[9 /* IVH sum score */] <3.5f ?

0.9f :

0.82608694f)));

return pred;

} // constant pool size = 31B, number of visited nodes = 6, static init size = 30B

// {11100000 00000000 00000000 00000000}

public static final byte[] GRPSPLIT0 = new byte[] {7, 0, 0, 0};

}

class DRF_4_Tree_3_class_1 {

static final double score0(double[] data) {

double pred = (data[3 /* Fisher */] <3.5f ?

(Double.isNaN(data[10]) || data[10 /* Post-treatment Bicaudate Index */] <0.175f ?

(data[12 /* ASA SCORE */] <1.5f ?

0.0f :

(Double.isNaN(data[10]) || data[10 /* Post-treatment Bicaudate Index */] <0.13535155f ?

0.0f :

0.04761905f)) :

0.13636364f) :

(Double.isNaN(data[25 /* DCI */]) || !GenModel.bitSetIsInRange(32, 0, data[25]) || (GenModel.bitSetIsInRange(32, 0, data[25]) && !GenModel.bitSetContains(GRPSPLIT0, 32, 0, data[25])) ?

(Double.isNaN(data[17 /* EVD */]) || !GenModel.bitSetIsInRange(32, 0, data[17]) || (GenModel.bitSetIsInRange(32, 0, data[17]) && !GenModel.bitSetContains(GRPSPLIT1, 32, 0, data[17])) ?

0.030303031f :

0.3f) :

(!Double.isNaN(data[6 /* ICH */]) && (GenModel.bitSetIsInRange(32, 0, data[6]) && !GenModel.bitSetContains(GRPSPLIT2, 32, 0, data[6])) ?

0.25f :

0.45f)));

return pred;

} // constant pool size = 45B, number of visited nodes = 7, static init size = 90B

// {00010000 00000000 00000000 00000000}

public static final byte[] GRPSPLIT0 = new byte[] {8, 0, 0, 0};

// {01000000 00000000 00000000 00000000}

public static final byte[] GRPSPLIT1 = new byte[] {2, 0, 0, 0};

// {01000000 00000000 00000000 00000000}

public static final byte[] GRPSPLIT2 = new byte[] {2, 0, 0, 0};

}

class DRF_4_Forest_4 {

public static void score0(double[] fdata, double[] preds) {

preds[1] += DRF_4_Tree_4_class_0.score0(fdata);

preds[2] += DRF_4_Tree_4_class_1.score0(fdata);

}

}

class DRF_4_Tree_4_class_0 {

static final double score0(double[] data) {

double pred = (Double.isNaN(data[14]) || data[14 /* NIHSS on admission */] <1.5f ?

(Double.isNaN(data[24 /* Post-intervention ICU */]) || !GenModel.bitSetIsInRange(32, 0, data[24]) || (GenModel.bitSetIsInRange(32, 0, data[24]) && !GenModel.bitSetContains(GRPSPLIT0, 32, 0, data[24])) ?

(Double.isNaN(data[20 /* Aneurysm location */]) || !GenModel.bitSetIsInRange(32, 0, data[20]) || (GenModel.bitSetIsInRange(32, 0, data[20]) && !GenModel.bitSetContains(GRPSPLIT1, 32, 0, data[20])) ?

0.85294116f :

1.0f) :

1.0f) :

(Double.isNaN(data[10]) || data[10 /* Post-treatment Bicaudate Index */] <0.225f ?

0.8727273f :

0.53846157f));

return pred;

} // constant pool size = 28B, number of visited nodes = 4, static init size = 60B

// {10000000 00000000 00000000 00000000}

public static final byte[] GRPSPLIT0 = new byte[] {1, 0, 0, 0};

// {11101011 11111000 00000000 00000000}

public static final byte[] GRPSPLIT1 = new byte[] {-41, 31, 0, 0};

}

class DRF_4_Tree_4_class_1 {

static final double score0(double[] data) {

double pred = (Double.isNaN(data[14]) || data[14 /* NIHSS on admission */] <1.5f ?

(!Double.isNaN(data[24 /* Post-intervention ICU */]) && (GenModel.bitSetIsInRange(32, 0, data[24]) && !GenModel.bitSetContains(GRPSPLIT0, 32, 0, data[24])) ?

0.0f :

(!Double.isNaN(data[20 /* Aneurysm location */]) && (GenModel.bitSetIsInRange(32, 0, data[20]) && !GenModel.bitSetContains(GRPSPLIT1, 32, 0, data[20])) ?

0.0f :

0.14705883f)) :

(Double.isNaN(data[10]) || data[10 /* Post-treatment Bicaudate Index */] <0.225f ?

0.12727273f :

0.46153846f));

return pred;

} // constant pool size = 28B, number of visited nodes = 4, static init size = 60B

// {01000000 00000000 00000000 00000000}

public static final byte[] GRPSPLIT0 = new byte[] {2, 0, 0, 0};

// {00010100 00000000 00000000 00000000}

public static final byte[] GRPSPLIT1 = new byte[] {40, 0, 0, 0};

}

class DRF_4_Forest_5 {

public static void score0(double[] fdata, double[] preds) {

preds[1] += DRF_4_Tree_5_class_0.score0(fdata);

preds[2] += DRF_4_Tree_5_class_1.score0(fdata);

}

}

class DRF_4_Tree_5_class_0 {

static final double score0(double[] data) {

double pred = (!Double.isNaN(data[31 /* Other Infections */]) && (GenModel.bitSetIsInRange(32, 0, data[31]) && !GenModel.bitSetContains(GRPSPLIT0, 32, 0, data[31])) ?

(Double.isNaN(data[20 /* Aneurysm location */]) || !GenModel.bitSetIsInRange(32, 0, data[20]) || (GenModel.bitSetIsInRange(32, 0, data[20]) && !GenModel.bitSetContains(GRPSPLIT1, 32, 0, data[20])) ?

(!Double.isNaN(data[17 /* EVD */]) && (GenModel.bitSetIsInRange(32, 0, data[17]) && !GenModel.bitSetContains(GRPSPLIT2, 32, 0, data[17])) ?

0.5f :

0.8695652f) :

0.95f) :

(!Double.isNaN(data[7 /* IVH */]) && (GenModel.bitSetIsInRange(32, 0, data[7]) && !GenModel.bitSetContains(GRPSPLIT3, 32, 0, data[7])) ?

(data[0 /* Age at SAH */] <55.5f ?

0.96f :

0.85714287f) :

1.0f));

return pred;

} // constant pool size = 42B, number of visited nodes = 5, static init size = 120B

// {10000000 00000000 00000000 00000000}

public static final byte[] GRPSPLIT0 = new byte[] {1, 0, 0, 0};

// {11001101 11111000 00000000 00000000}

public static final byte[] GRPSPLIT1 = new byte[] {-77, 31, 0, 0};

// {10000000 00000000 00000000 00000000}

public static final byte[] GRPSPLIT2 = new byte[] {1, 0, 0, 0};

// {10000000 00000000 00000000 00000000}

public static final byte[] GRPSPLIT3 = new byte[] {1, 0, 0, 0};

}

class DRF_4_Tree_5_class_1 {

static final double score0(double[] data) {

double pred = (Double.isNaN(data[31 /* Other Infections */]) || !GenModel.bitSetIsInRange(32, 0, data[31]) || (GenModel.bitSetIsInRange(32, 0, data[31]) && !GenModel.bitSetContains(GRPSPLIT0, 32, 0, data[31])) ?

(Double.isNaN(data[20 /* Aneurysm location */]) || !GenModel.bitSetIsInRange(32, 0, data[20]) || (GenModel.bitSetIsInRange(32, 0, data[20]) && !GenModel.bitSetContains(GRPSPLIT1, 32, 0, data[20])) ?

(Double.isNaN(data[9]) || data[9 /* IVH sum score */] <2.5f ?

0.0f :

0.05f) :

0.15384616f) :

(Double.isNaN(data[18 /* Days with EVD */]) || !GenModel.bitSetIsInRange(32, 0, data[18]) || (GenModel.bitSetIsInRange(32, 0, data[18]) && !GenModel.bitSetContains(GRPSPLIT2, 32, 0, data[18])) ?

0.083333336f :

0.41379312f));

return pred;

} // constant pool size = 33B, number of visited nodes = 4, static init size = 90B

// {01000000 00000000 00000000 00000000}

public static final byte[] GRPSPLIT0 = new byte[] {2, 0, 0, 0};

// {10000100 00001000 00000000 00000000}

public static final byte[] GRPSPLIT1 = new byte[] {33, 16, 0, 0};

// {01000000 00000000 00000000 00000000}

public static final byte[] GRPSPLIT2 = new byte[] {2, 0, 0, 0};

}

class DRF_4_Forest_6 {

public static void score0(double[] fdata, double[] preds) {

preds[1] += DRF_4_Tree_6_class_0.score0(fdata);

preds[2] += DRF_4_Tree_6_class_1.score0(fdata);

}

}

class DRF_4_Tree_6_class_0 {

static final double score0(double[] data) {

double pred = (data[3 /* Fisher */] <3.5f ?

(!Double.isNaN(data[8 /* Treatment timing from symptoms onset */]) && (GenModel.bitSetIsInRange(32, 0, data[8]) && !GenModel.bitSetContains(GRPSPLIT0, 32, 0, data[8])) ?

0.9f :

1.0f) :

(Double.isNaN(data[15]) || data[15 /* NIHSS Motor on admission */] <1.0f ?

(data[13 /* KPS on admission */] <64.5f ?

0.8f :

0.875f) :

(Double.isNaN(data[9]) || data[9 /* IVH sum score */] <4.5f ?

0.875f :

0.55f)));

return pred;

} // constant pool size = 27B, number of visited nodes = 5, static init size = 30B

// {10010000 00000000 00000000 00000000}

public static final byte[] GRPSPLIT0 = new byte[] {9, 0, 0, 0};

}

class DRF_4_Tree_6_class_1 {

static final double score0(double[] data) {

double pred = (data[3 /* Fisher */] <3.5f ?

(Double.isNaN(data[8 /* Treatment timing from symptoms onset */]) || !GenModel.bitSetIsInRange(32, 0, data[8]) || (GenModel.bitSetIsInRange(32, 0, data[8]) && !GenModel.bitSetContains(GRPSPLIT0, 32, 0, data[8])) ?

0.0f :

0.1f) :

(Double.isNaN(data[15]) || data[15 /* NIHSS Motor on admission */] <1.0f ?

(data[13 /* KPS on admission */] <64.5f ?

0.2f :

0.125f) :

(Double.isNaN(data[9]) || data[9 /* IVH sum score */] <4.5f ?

0.125f :

0.45f)));

return pred;

} // constant pool size = 27B, number of visited nodes = 5, static init size = 30B

// {01100000 00000000 00000000 00000000}

public static final byte[] GRPSPLIT0 = new byte[] {6, 0, 0, 0};

}

class DRF_4_Forest_7 {

public static void score0(double[] fdata, double[] preds) {

preds[1] += DRF_4_Tree_7_class_0.score0(fdata);

preds[2] += DRF_4_Tree_7_class_1.score0(fdata);

}

}

class DRF_4_Tree_7_class_0 {

static final double score0(double[] data) {

double pred = (Double.isNaN(data[14]) || data[14 /* NIHSS on admission */] <1.5f ?

(Double.isNaN(data[9]) || data[9 /* IVH sum score */] <0.5f ?

1.0f :

0.875f) :

(Double.isNaN(data[11]) || data[11 /* mRS on admission */] <4.5f ?

(Double.isNaN(data[31 /* Other Infections */]) || !GenModel.bitSetIsInRange(32, 0, data[31]) || (GenModel.bitSetIsInRange(32, 0, data[31]) && !GenModel.bitSetContains(GRPSPLIT0, 32, 0, data[31])) ?

0.5f :

0.8f) :

0.9310345f));

return pred;

} // constant pool size = 23B, number of visited nodes = 4, static init size = 30B

// {10000000 00000000 00000000 00000000}

public static final byte[] GRPSPLIT0 = new byte[] {1, 0, 0, 0};

}

class DRF_4_Tree_7_class_1 {

static final double score0(double[] data) {

double pred = (Double.isNaN(data[14]) || data[14 /* NIHSS on admission */] <1.5f ?

(Double.isNaN(data[9]) || data[9 /* IVH sum score */] <0.5f ?

0.0f :

0.125f) :

(Double.isNaN(data[11]) || data[11 /* mRS on admission */] <4.5f ?

(!Double.isNaN(data[31 /* Other Infections */]) && (GenModel.bitSetIsInRange(32, 0, data[31]) && !GenModel.bitSetContains(GRPSPLIT0, 32, 0, data[31])) ?

0.2f :

0.5f) :

0.06896552f));

return pred;

} // constant pool size = 23B, number of visited nodes = 4, static init size = 30B

// {01000000 00000000 00000000 00000000}

public static final byte[] GRPSPLIT0 = new byte[] {2, 0, 0, 0};

}

class DRF_4_Forest_8 {

public static void score0(double[] fdata, double[] preds) {

preds[1] += DRF_4_Tree_8_class_0.score0(fdata);

preds[2] += DRF_4_Tree_8_class_1.score0(fdata);

}

}

class DRF_4_Tree_8_class_0 {

static final double score0(double[] data) {

double pred = (Double.isNaN(data[10]) || data[10 /* Post-treatment Bicaudate Index */] <0.225f ?

(!Double.isNaN(data[17 /* EVD */]) && (GenModel.bitSetIsInRange(32, 0, data[17]) && !GenModel.bitSetContains(GRPSPLIT0, 32, 0, data[17])) ?

0.7307692f :

(Double.isNaN(data[20 /* Aneurysm location */]) || !GenModel.bitSetIsInRange(32, 0, data[20]) || (GenModel.bitSetIsInRange(32, 0, data[20]) && !GenModel.bitSetContains(GRPSPLIT1, 32, 0, data[20])) ?

(!Double.isNaN(data[7 /* IVH */]) && (GenModel.bitSetIsInRange(32, 0, data[7]) && !GenModel.bitSetContains(GRPSPLIT2, 32, 0, data[7])) ?

0.875f :

(Double.isNaN(data[0]) || data[0 /* Age at SAH */] <55.5f ?

1.0f :

0.95f)) :

1.0f)) :

0.61403507f);

return pred;

} // constant pool size = 37B, number of visited nodes = 5, static init size = 90B

// {10000000 00000000 00000000 00000000}

public static final byte[] GRPSPLIT0 = new byte[] {1, 0, 0, 0};

// {01101011 11111000 00000000 00000000}

public static final byte[] GRPSPLIT1 = new byte[] {-42, 31, 0, 0};

// {10000000 00000000 00000000 00000000}

public static final byte[] GRPSPLIT2 = new byte[] {1, 0, 0, 0};

}

class DRF_4_Tree_8_class_1 {

static final double score0(double[] data) {

double pred = (Double.isNaN(data[10]) || data[10 /* Post-treatment Bicaudate Index */] <0.225f ?

(Double.isNaN(data[17 /* EVD */]) || !GenModel.bitSetIsInRange(32, 0, data[17]) || (GenModel.bitSetIsInRange(32, 0, data[17]) && !GenModel.bitSetContains(GRPSPLIT0, 32, 0, data[17])) ?

(Double.isNaN(data[4]) || data[4 /* Hunt-Hess */] <2.5f ?

(Double.isNaN(data[7 /* IVH */]) || !GenModel.bitSetIsInRange(32, 0, data[7]) || (GenModel.bitSetIsInRange(32, 0, data[7]) && !GenModel.bitSetContains(GRPSPLIT1, 32, 0, data[7])) ?

0.0f :

0.05f) :

0.08571429f) :

0.26923078f) :

0.3859649f);

return pred;

} // constant pool size = 28B, number of visited nodes = 4, static init size = 60B

// {01000000 00000000 00000000 00000000}

public static final byte[] GRPSPLIT0 = new byte[] {2, 0, 0, 0};

// {01000000 00000000 00000000 00000000}

public static final byte[] GRPSPLIT1 = new byte[] {2, 0, 0, 0};

}

class DRF_4_Forest_9 {

public static void score0(double[] fdata, double[] preds) {

preds[1] += DRF_4_Tree_9_class_0.score0(fdata);

preds[2] += DRF_4_Tree_9_class_1.score0(fdata);

}

}

class DRF_4_Tree_9_class_0 {

static final double score0(double[] data) {

double pred = (Double.isNaN(data[7 /* IVH */]) || !GenModel.bitSetIsInRange(32, 0, data[7]) || (GenModel.bitSetIsInRange(32, 0, data[7]) && !GenModel.bitSetContains(GRPSPLIT0, 32, 0, data[7])) ?

(!Double.isNaN(data[18 /* Days with EVD */]) && (GenModel.bitSetIsInRange(32, 0, data[18]) && !GenModel.bitSetContains(GRPSPLIT1, 32, 0, data[18])) ?

(Double.isNaN(data[0]) || data[0 /* Age at SAH */] <61.5f ?

0.7419355f :

0.3809524f) :

(data[4 /* Hunt-Hess */] <2.5f ?

1.0f :

0.875f)) :

(data[12 /* ASA SCORE */] <1.5f ?

0.93333334f :

1.0f));

return pred;

} // constant pool size = 32B, number of visited nodes = 5, static init size = 60B

// {10000000 00000000 00000000 00000000}

public static final byte[] GRPSPLIT0 = new byte[] {1, 0, 0, 0};

// {10000000 00000000 00000000 00000000}

public static final byte[] GRPSPLIT1 = new byte[] {1, 0, 0, 0};

}

class DRF_4_Tree_9_class_1 {

static final double score0(double[] data) {

double pred = (!Double.isNaN(data[7 /* IVH */]) && (GenModel.bitSetIsInRange(32, 0, data[7]) && !GenModel.bitSetContains(GRPSPLIT0, 32, 0, data[7])) ?

(Double.isNaN(data[31 /* Other Infections */]) || !GenModel.bitSetIsInRange(32, 0, data[31]) || (GenModel.bitSetIsInRange(32, 0, data[31]) && !GenModel.bitSetContains(GRPSPLIT1, 32, 0, data[31])) ?

0.0f :

0.08695652f) :

(data[5 /* WFNS */] <1.5f ?

0.15f :

(!Double.isNaN(data[17 /* EVD */]) && (GenModel.bitSetIsInRange(32, 0, data[17]) && !GenModel.bitSetContains(GRPSPLIT2, 32, 0, data[17])) ?

0.12f :

0.41025642f)));

return pred;

} // constant pool size = 33B, number of visited nodes = 4, static init size = 90B

// {01000000 00000000 00000000 00000000}

public static final byte[] GRPSPLIT0 = new byte[] {2, 0, 0, 0};

// {01000000 00000000 00000000 00000000}

public static final byte[] GRPSPLIT1 = new byte[] {2, 0, 0, 0};

// {01000000 00000000 00000000 00000000}

public static final byte[] GRPSPLIT2 = new byte[] {2, 0, 0, 0};

}

class DRF_4_Forest_10 {

public static void score0(double[] fdata, double[] preds) {

preds[1] += DRF_4_Tree_10_class_0.score0(fdata);

preds[2] += DRF_4_Tree_10_class_1.score0(fdata);

}

}

class DRF_4_Tree_10_class_0 {

static final double score0(double[] data) {

double pred = (Double.isNaN(data[9]) || data[9 /* IVH sum score */] <4.5f ?

(Double.isNaN(data[14]) || data[14 /* NIHSS on admission */] <1.5f ?

(!Double.isNaN(data[7 /* IVH */]) && (GenModel.bitSetIsInRange(32, 0, data[7]) && !GenModel.bitSetContains(GRPSPLIT0, 32, 0, data[7])) ?

0.88461536f :

1.0f) :

(!Double.isNaN(data[17 /* EVD */]) && (GenModel.bitSetIsInRange(32, 0, data[17]) && !GenModel.bitSetContains(GRPSPLIT1, 32, 0, data[17])) ?

0.55f :

0.8918919f)) :

0.66101694f);

return pred;

} // constant pool size = 28B, number of visited nodes = 4, static init size = 60B

// {10000000 00000000 00000000 00000000}

public static final byte[] GRPSPLIT0 = new byte[] {1, 0, 0, 0};

// {10000000 00000000 00000000 00000000}

public static final byte[] GRPSPLIT1 = new byte[] {1, 0, 0, 0};

}

class DRF_4_Tree_10_class_1 {

static final double score0(double[] data) {

double pred = (Double.isNaN(data[9]) || data[9 /* IVH sum score */] <4.5f ?

(Double.isNaN(data[14]) || data[14 /* NIHSS on admission */] <1.5f ?

(Double.isNaN(data[7 /* IVH */]) || !GenModel.bitSetIsInRange(32, 0, data[7]) || (GenModel.bitSetIsInRange(32, 0, data[7]) && !GenModel.bitSetContains(GRPSPLIT0, 32, 0, data[7])) ?

0.0f :

0.115384616f) :

(Double.isNaN(data[17 /* EVD */]) || !GenModel.bitSetIsInRange(32, 0, data[17]) || (GenModel.bitSetIsInRange(32, 0, data[17]) && !GenModel.bitSetContains(GRPSPLIT1, 32, 0, data[17])) ?

0.10810811f :

0.45f)) :

0.33898306f);

return pred;

} // constant pool size = 28B, number of visited nodes = 4, static init size = 60B

// {01000000 00000000 00000000 00000000}

public static final byte[] GRPSPLIT0 = new byte[] {2, 0, 0, 0};

// {01000000 00000000 00000000 00000000}

public static final byte[] GRPSPLIT1 = new byte[] {2, 0, 0, 0};

}

class DRF_4_Forest_11 {

public static void score0(double[] fdata, double[] preds) {

preds[1] += DRF_4_Tree_11_class_0.score0(fdata);

preds[2] += DRF_4_Tree_11_class_1.score0(fdata);

}

}

class DRF_4_Tree_11_class_0 {

static final double score0(double[] data) {

double pred = (Double.isNaN(data[10]) || data[10 /* Post-treatment Bicaudate Index */] <0.225f ?

(data[4 /* Hunt-Hess */] <1.5f ?

1.0f :

(Double.isNaN(data[28 /* Fever onset */]) || !GenModel.bitSetIsInRange(32, 0, data[28]) || (GenModel.bitSetIsInRange(32, 0, data[28]) && !GenModel.bitSetContains(GRPSPLIT0, 32, 0, data[28])) ?

(!Double.isNaN(data[31 /* Other Infections */]) && (GenModel.bitSetIsInRange(32, 0, data[31]) && !GenModel.bitSetContains(GRPSPLIT1, 32, 0, data[31])) ?

0.7941176f :

0.95f) :

1.0f)) :

0.61403507f);

return pred;

} // constant pool size = 28B, number of visited nodes = 4, static init size = 60B

// {10100000 00000000 00000000 00000000}

public static final byte[] GRPSPLIT0 = new byte[] {5, 0, 0, 0};

// {10000000 00000000 00000000 00000000}

public static final byte[] GRPSPLIT1 = new byte[] {1, 0, 0, 0};

}
